# Temporal Patterns in Preterm Birth Subtypes and Perinatal Survival, 2000–2023: Population‐Based, Repeated Cross‐Sectional Time‐Series

**DOI:** 10.1111/1471-0528.70166

**Published:** 2026-01-26

**Authors:** Ka Wang Cheung, Tiffany Sin‐Tung Au, Tat On Chan, Annie Shuk Yi Hui, Po Lam So, Daniel Wong, Tsz Kin Lo, Wai Lam Lau, Choi Wah Kong, Teresa Wei Ling Ma, Ying Rong Li, Mimi Tin‐Yan Seto

**Affiliations:** ^1^ Department of Obstetrics and Gynaecology Queen Mary Hospital, The University of Hong Kong Hong Kong Hong Kong SAR China; ^2^ Department of Obstetrics and Gynaecology The University of Hong Kong— Shenzhen Hospital Shenzhen China; ^3^ Department of Obstetrics and Gynaecology Prince of Wales Hospital, the Chinese University of Hong Kong Hong Kong Hong Kong SAR China; ^4^ Department of Obstetrics and Gynaecology Tuen Mun Hospital Hong Kong Hong Kong SAR China; ^5^ Department of Obstetrics and Gynaecology Pamela Youde Nethersole Eastern Hospital Hong Kong Hong Kong SAR China; ^6^ Department of Obstetrics and Gynaecology Princess Margaret Hospital Hong Kong Hong Kong SAR China; ^7^ Department of Obstetrics and Gynaecology Kwong Wah Hospital Hong Kong Hong Kong SAR China; ^8^ Department of Obstetrics and Gynaecology United Christian Hospital Hong Kong Hong Kong SAR China; ^9^ Department of Obstetrics and Gynaecology Queen Elizabeth Hospital Hong Kong Hong Kong SAR China

**Keywords:** low birth weight, mortality, prematurity, preterm labour, stillbirth

## Abstract

**Objective:**

Evaluate the rate and trend of preterm birth (PTB) and the associated perinatal survival in Hong Kong.

**Design:**

Population‐based, repeated cross‐sectional time series.

**Setting:**

All public maternity hospitals.

**Population:**

845 640 singleton and 26 748 twin deliveries from 2000 to 2023.

**Methods:**

Two analyses were performed (i) spontaneous PTB (sPTB) vs. iatrogenic PTB (iPTB), (ii) spontaneous PTB with no preterm prelabour rupture of membranes (PPROM) (sPTB‐noPPROM) versus. PTB following PPROM (PTB‐PPROM) vs. iPTB.

**Main Outcome Measures:**

PTB rate and perinatal survival.

**Results:**

Simply dividing PTB into sPTB and iPTB demonstrated increasing PTB rates for both types amongst singleton and twin pregnancy. Subclassification of sPTB into sPTB‐noPPROM and PTB‐PPROM demonstrated a downward trend in sPTB‐noPPROM while PTB‐PPROM increased significantly (singleton: sPTB‐noPPROM average annual percent change (AAPC) −0.96, 95% CI −1.67 to −0.41; PTB‐PPROM AAPC 3.4, 95% CI 2.89 to 4.18; iPTB AAPC 2.01, 95% CI 1.57 to 2.45; twin: sPTB‐noPPROM AAPC −2.62, 95% CI −4.09 to −1.58; PTB‐PPROM AAPC 3.05, 95% CI 2.46 to 3.72; iPTB AAPC 2.75, 95% CI 1.82 to 3.69). PTB‐PPROM and iPTB were the leading causes of PTB for singleton and twin pregnancy, respectively. Hypertensive disorders were the predominant causes of iPTB for both singleton and twin pregnancy while iPTB indicated by maternal illnesses reduced over time. Reduction of neonatal mortality rates were noted amongst iPTB for singleton < 37 weeks and twin pregnancies at 34^+0^–36^+6^ weeks. The rate of stillbirth and perinatal mortality also reduced amongst PTB of twin pregnancies.

**Conclusions:**

Perinatal survival improved despite increasing PTB rates. The trend of PTB should be interpreted in the context of perinatal health indicators, and categorisation of PTB could aid with truly reflecting and explaining the trends and patterns of PTB within a population.

## 1 Introduction

Although significant effort has been made to reduce the burden of preterm birth (PTB), a systematic analysis found no change in the PTB rate globally or across different regions in the last decade, with an estimated global PTB rate of 9.8% in 2010 and 9.9% in 2020 [1] PTB rate in high income countries should not be overlooked. Amongst high income countries, an increase in PTB rates was observed in Greece, Bahrain and the US between 2010 and 2020 (Greece: 11.2% to 11.6%, Bahrain: 9.9% to 11.3%, US: 9.7% to 10%) [1] Possible reasons for the increased PTB rate include a higher proportion of women with advanced maternal age, an increase in the use of assisted reproductive techniques as well as the incidence of multiple pregnancy [2]. In Hong Kong, PTB is one of the leading causes of neonatal death, accounting for 29% of neonatal mortality between 1980 and 2017 [3]. An earlier local, single institute cohort study found a stable PTB rate of 6.5% in singleton pregnancy between 1995 and 2011 [4].

Many previous reports did not differentiate between different types of PTB, divide PTB into different gestational ages, or correlate with perinatal mortalities. Evaluating the overall PTB rate alone oversimplifies the problem of PTB. Stratification into factors based on aetiology or antecedent events promotes a more nuanced analysis and is more likely to produce advancements in antenatal care. PTB is generally classified into spontaneous or iatrogenic [5]. However, there is no consensus on how to classify PTB following preterm prelabour rupture of membranes (PPROM) amongst previous reports. Some defined it as spontaneous, some divided it into spontaneous or iatrogenic depending on the labour onset, and some allocated it to a separate entity.

We hypothesise that the on‐going revolution of modern obstetric healthcare has influenced the trend of PTB and the perinatal outcomes amongst PTB infants. Therefore, in this study, we aim to evaluate, (1) the rate of PTB in all public hospitals in Hong Kong, (2) the trend of different types of PTB (using different classifications: spontaneous PTB/iatrogenic PTB/PTB following rupture of membranes) across various gestational ages (late, moderate, very, and extreme PTB), and (3) the associated perinatal survival (stillbirth, perinatal mortality, and neonatal mortality) amongst singleton and twin pregnancy between 2000 and 2023.

## 2 Materials and Methods

A population‐based, repeated cross‐sectional time‐series study was carried out, involving all eight public maternity hospitals in Hong Kong. Clinical information was prospectively coded in the computerised clinical database utilised by the Hospital Authority, Hong Kong, to capture all healthcare data in the public settings. All delivery episodes recorded in these units between 2000 and 2023 were extracted. Data were retrieved via the Clinical Data Analysis and Reporting System. Details on the onset of labour were collected. The gestational age was estimated based on the date of the last menstrual period and/or by the ultrasound biometry in the first or second trimester of pregnancy. We excluded cases that had missing data on the gestational age at delivery, delivery before 24 weeks of gestation, and triplet and higher order multiple pregnancies.

Outcomes were defined as follows:

- PTB: livebirth between 24^+0^ and 36^+6^ weeks
- Timing of PTB
  ○ Late PTB: livebirth between 34^+0^ and 36^+6^ weeks
  ○ Moderate PTB: livebirth between 32^+0^ and 33^+6^ weeks.
  ○ Very PTB: livebirth between 28^+0^ and 31^+6^ weeks
  ○ Extreme PTB: livebirth between 24^+0^ and 27^+6^ weeks
- Types of PTB.
  ○ Spontaneous PTB including PTB following PPROM (sPTB): birth after spontaneous onset of labour or PPROM, which could be further subdivided into
    - Spontaneous PTB with no PPROM (sPTB‐noPPROM): birth after spontaneous onset of labour, excluding any births after PPROM.
    - PTB following PPROM (PTB‐PPROM): any birth after PPROM (including spontaneous onset or after induction of labour)
  ○ Iatrogenic PTB (iPTB): birth without spontaneous onset of labour (i.e., induction of labour or Caesarean section prior to spontaneous onset of labour) (no deliveries with PPROM were included)
- Stillbirth rate: the number of babies born with no signs of life at or after 24^+0^ gestational weeks, or with a birth weight of more than 500 g when the gestation age is uncertain, per 1000 total births
- Perinatal mortality rate: the number of stillbirths and deaths in the first week of life per 1000 total births
- Neonatal mortality rate: the number of deaths during the first 28 completed days of life per 1000 livebirths

Ethical approval was obtained from the institutional review board of all units and informed consent was not required due to the retrospective design of this study. This study was reported following the Strengthening the Reporting of Observational Studies in Epidemiology (STROBE) reporting guideline.

### 2.1 Statistical Analysis

PTB rates amongst singleton and twin pregnancies were reported separately. The PTB rate was calculated as the number of PTB per 100 livebirths, as stillbirths were commonly delivered preterm and may falsely inflate the PTB rate. To facilitate comparisons with other statistics that include stillbirths in PTB calculations, a supplementary analysis with the PTB rate calculated as per 100 total births (i.e., PTB rate = PTB (livebirth and stillbirth)/total births) was also performed [6]. For twin pregnancies, the rate of PTB was calculated by the number of pregnancies and included pregnancies that resulted in one livebirth and one stillbirth. All PTB deliveries with any of the following ICD‐9 diagnosis codes: 658.10:0, 658.10:1, 658:10:2, 658.11:0, 658.13:0, were identified as a delivery following PPROM. The rate of PTB overall, amongst different types of PTB (sPTB, sPTB‐noPPROM, PTB‐PPROM and iPTB), as well as across different gestational ages (late, moderate, very and extreme) was calculated. PTB with missing information on labour onset was excluded from the calculations of different types of PTB. Two separate analyses were performed (i) sPTB (including PTB following PPROM) versus iPTB, and (ii) sPTB‐noPPROM versus PTB‐PPROM versus iPTB. The rate of stillbirth, perinatal mortality and neonatal mortality amongst births at < 37 weeks of gestation was calculated as defined above, and the number of deliveries was used for twin pregnancies. Stillbirths from twin pregnancies that resulted in one livebirth and one stillbirth were excluded from calculations for neonatal mortality.

Indications for iPTB were categorised into the groups based on ICD‐9 diagnosis codes Table S1. Deliveries with no diagnosis codes recorded that could explain the indication for induction of labour or caesarean section were excluded from the calculations. To avoid duplication amongst iPTB with more than one relevant ICD‐9 diagnosis code, an order of importance was designated to the groups of indications, similar to previous report [7]. The first group was considered to have the highest likelihood of causing an iPTB and thus overrode the others. The groups of indication in order were hypertensive disorders, antepartum haemorrhage, chorioamnionitis, abnormal cardiotocogram, twin complications, intrauterine growth restriction, oligohydramnios, red cell isoimmunisation, cholestasis, maternal disease, and twin pregnancy.

To analyse the trend of PTB, stillbirth, perinatal mortality, neonatal mortality and indication for iPTB in the study period, % change between 2000 and 2023, as well as the average annual percent change (AAPC) throughout the study period with 95% confidence intervals (CIs) were calculated using the approach proposed by Kim et al. [8, 9], using the Joinpoint Regression Program (Version 5.3.0.0, November 2024; National Cancer Institute) [10]. Rates of each year were natural‐log transformed, while constant variance (homoscedasticity) and uncorrelated error were assumed for modelling. Weighted Bayesian Information Criterion was used for model selection. *p* < 0.05 was considered statistically significant. Triplet, or high‐order multiple pregnancies, deliveries before 24 weeks of gestation and those with no data on the gestational age at delivery were excluded from all analyses.

## 3 Results

There were 875 372 deliveries from 2000 to 2023 across all eight public maternity hospitals in Hong Kong. 720 deliveries with no data on gestational age at delivery, 1507 deliveries before 24 weeks of gestation, and 757 deliveries from triplet or higher order multiple pregnancies were excluded, leaving 845 640 singleton and 13 374 (26 748 deliveries) twin pregnancies for final analysis. There were 2545 singleton stillbirths, and 35 twin pregnancies resulted in stillbirth of both twins. 147 twin pregnancies had one stillbirth and one livebirth. Figure S1 shows the identification, inclusion, and exclusion of study subjects. 17 singleton PTB had missing information on the labour onset and were excluded from the analysis on the types of PTB. 586 singleton iPTB had missing information on the underlying cause for iPTB. Table S2 shows the basic demographics of the included subjects. Overall, approximately 91% were Chinese and 52% were nulliparous.

### 3.1 Singleton Pregnancy

**FIGURE 1:**
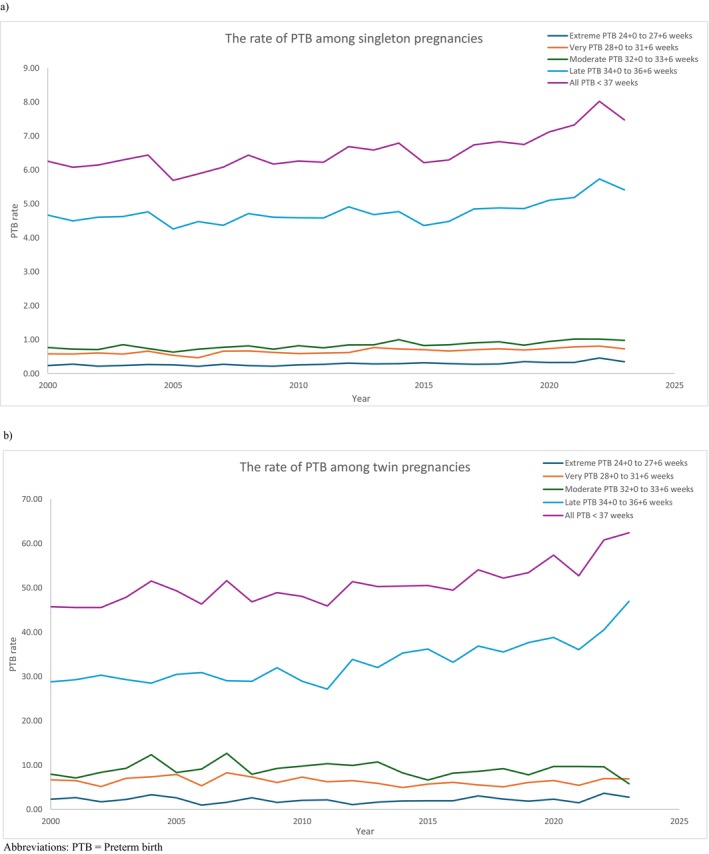
The rate of preterm birth between 2000 and 2023 amongst (a) singleton, (b) twin pregnancies. PTB, Preterm birth.

Table 1, Table S3 and Figure 1a show the numbers and rates of PTB across various gestations amongst singleton pregnancies from 2000 to 2023. The overall PTB rate rose significantly from 6.26% in 2000 to 7.47% in 2023 (average annual percent change (AAPC) 1.06, 95% CI 0.59 to 1.44). The rates of extreme, very, moderate and late PTB also increased significantly (Table S3). Supplementary analysis evaluating the rate of PTB per total births (i.e., stillbirth included) demonstrated similar trends; the overall PTB rate rose significantly from 6.46% to 7.71% (AAPC 1.08, 95% CI 0.61 to 1.44). (Table S4).

**TABLE 1:** The numbers and rates of preterm birth across various gestations amongst singleton and twin pregnancies from 2000 to 2023.

Singleton pregnancy																												
	Total	2000	2001	2002	2003	2004	2005	2006	2007	2008	2009	2010	2011	2012	2013	2014	2015	2016	2017	2018	2019	2020	2021	2022	2023	% Change 2000–2023	Average annual percent change (95% CI)	*p*
	*n* = 843 095	*n* = 38 362	*n* = 35 312	*n* = 36 315	*n* = 34 736	*n* = 36 754	*n* = 40 244	*n* = 39 383	*n* = 38 381	*n* = 31 542	*n* = 39 727	*n* = 41 721	*n* = 44 372	*n* = 43 181	*n* = 35 708	*n* = 38 713	*n* = 38 231	*n* = 39 160	*n* = 36 461	*n* = 34 209	*n* = 32 908	*n* = 25 885	*n* = 22 703	*n* = 19 097	*n* = 19 990			
Overall	54 389 (6.45)	2400 (6.26)	2147 (6.08)	2231 (6.14)	2187 (6.30)	2366 (6.44)	2291 (5.69)	2317 (5.88)	2335 (6.08)	2030 (6.44)	2452 (6.17)	2612 (6.26)	2763 (6.23)	2888 (6.69)	2352 (6.59)	2629 (6.79)	2376 (6.21)	2465 (6.29)	2457 (6.74)	2338 (6.83)	2221 (6.75)	1843 (7.12)	1663 (7.33)	1532 (8.02)	1494 (7.47)	19.46	1.06 (0.59, 1.44)	< 0.001
sPTB	39 596 (4.70)	1728 (4.51)	1586 (4.49)	1655 (4.56)	1618 (4.66)	1776 (4.83)	1649 (4.10)	1749 (4.44)	1728 (4.50)	1564 (4.96)	1800 (4.53)	1997 (4.79)	2119 (4.78)	2188 (5.07)	1731 (4.85)	1939 (5.01)	1707 (4.47)	1781 (4.55)	1742 (4.78)	1632 (4.77)	1556 (4.73)	1216 (4.70)	1106 (4.87)	1032 (5.41)	997 (4.99)	10.74	0.44 (0.11, 0.76)	0.008
sPTB‐noPPROM	18 973 (2.25)	1248 (3.25)	946 (2.68)	900 (2.48)	943 (2.72)	1001 (2.72)	904 (2.25)	969 (2.46)	888 (2.31)	780 (2.47)	914 (2.30)	899 (2.16)	923 (2.08)	951 (2.20)	727 (2.04)	801 (2.07)	676 (1.77)	703 (1.80)	694 (1.90)	642 (1.88)	598 (1.82)	502 (1.94)	483 (2.13)	449 (2.35)	432 (2.16)	−33.56	−0.96 (−1.67, −0.41)	< 0.001
PTB‐PPROM	20 623 (2.45)	480 (1.25)	640 (1.81)	755 (2.08)	675 (1.94)	775 (2.11)	745 (1.85)	780 (1.98)	840 (2.19)	784 (2.49)	886 (2.23)	1098 (2.63)	1196 (2.70)	1237 (2.87)	1004 (2.81)	1138 (2.94)	1031 (2.70)	1078 (2.75)	1048 (2.88)	990 (2.89)	958 (2.91)	714 (2.76)	623 (2.74)	583 (3.05)	565 (2.83)	125.92	3.46 (2.89, 4.18)	< 0.001
iPTB	14 776 (1.75)	671 (1.75)	559 (1.58)	574 (1.58)	568 (1.64)	590 (1.61)	640 (1.59)	568 (1.44)	605 (1.58)	465 (1.47)	652 (1.64)	614 (1.47)	644 (1.45)	699 (1.62)	621 (1.74)	689 (1.78)	669 (1.75)	683 (1.74)	715 (1.96)	706 (2.06)	665 (2.02)	627 (2.42)	557 (2.45)	500 (2.62)	495 (2.48)	41.59	2.01 (1.57, 2.45)	< 0.001

Twin pregnancy																												
	Total	2000	2001	2002	2003	2004	2005	2006	2007	2008	2009	2010	2011	2012	2013	2014	2015	2016	2017	2018	2019	2020	2021	2022	2023	% Change 2000–2023	Average annual percent change (95% CI)	*p*
	*n* = 13 339	*n* = 389	*n* = 338	*n* = 406	*n* = 355	*n* = 421	*n* = 456	*n* = 505	*n* = 616	*n* = 491	*n* = 691	*n* = 726	*n* = 832	*n* = 815	*n* = 662	*n* = 728	*n* = 663	*n* = 768	*n* = 686	*n* = 684	*n* = 640	*n* = 474	*n* = 330	*n* = 301	*n* = 362			
Overall	6739 (50.52)	178 (45.76)	154 (45.56)	185 (45.57)	170 (47.89)	217 (51.54)	225 (49.34)	234 (46.34)	318 (51.62)	230 (46.84)	338 (48.91)	349 (48.07)	382 (45.91)	419 (51.41)	333 (50.30)	367 (50.41)	335 (50.53)	380 (49.48)	371 (54.08)	357 (52.19)	342 (53.44)	272 (57.38)	174 (52.73)	183 (60.80)	226 (62.43)	36.44	1.15 (0.66, 1.57)	< 0.001
sPTB	3489 (26.16)	102 (26.22)	85 (25.15)	107 (26.35)	103 (29.01)	124 (29.45)	116 (25.49)	146 (28.97)	166 (26.95)	128 (26.07)	205 (29.67)	207 (28.51)	247 (29.69)	231 (28.34)	178 (26.89)	175 (24.04)	174 (26.24)	192 (25.00)	166 (24.20)	146 (21.35)	139 (21.72)	114 (24.05)	74 (22.42)	72 (23.92)	92 (25.41)	−3.08	−0.20 (−0.95, 0.39)	0.504
sPTB‐noPPROM	1393 (10.44)	74 (19.02)	45 (13.31)	54 (13.30)	51 (14.37)	66 (15.68)	67 (14.73)	70 (13.89)	65 (10.55)	49 (9.98)	79 (11.43)	83 (11.43)	99 (11.90)	78 (9.57)	64 (9.67)	54 (7.42)	64 (9.65)	69 (8.98)	58 (8.45)	46 (6.73)	35 (5.47)	40 (8.44)	23 (6.97)	25 (8.31)	35 (9.67)	−49.18	−2.62 (−4.09, −1.58)	< 0.001
PTB‐PPROM	2096 (15.72)	28 (7.20)	40 (11.83)	53 (13.05)	52 (14.65)	58 (13.78)	49 (10.77)	76 (15.08)	101 (16.40)	79 (16.09)	126 (18.23)	124 (17.08)	148 (17.79)	153 (18.77)	114 (17.22)	121 (16.62)	110 (16.59)	123 (16.02)	108 (15.74)	100 (14.62)	104 (16.25)	74 (15.61)	51 (15.45)	47 (15.61)	57 (15.75)	118.75	3.05 (2.46, 3.72)	< 0.001
iPTB	3250 (24.37)	76 (19.54)	69 (20.41)	78 (19.21)	67 (18.87)	93 (22.09)	109 (23.96)	88 (17.46)	152 (24.68)	102 (20.77)	133 (19.25)	142 (19.56)	135 (16.23)	188 (23.07)	155 (23.41)	192 (26.37)	161 (24.28)	188 (24.48)	205 (29.88)	211 (30.85)	203 (31.72)	158 (33.33)	100 (30.30)	111 (36.88)	134 (37.02)	89.47	2.75 (1.82, 3.69)	< 0.001

### 3.2 sPTB Versus iPTB—Singleton

**FIGURE 2:**
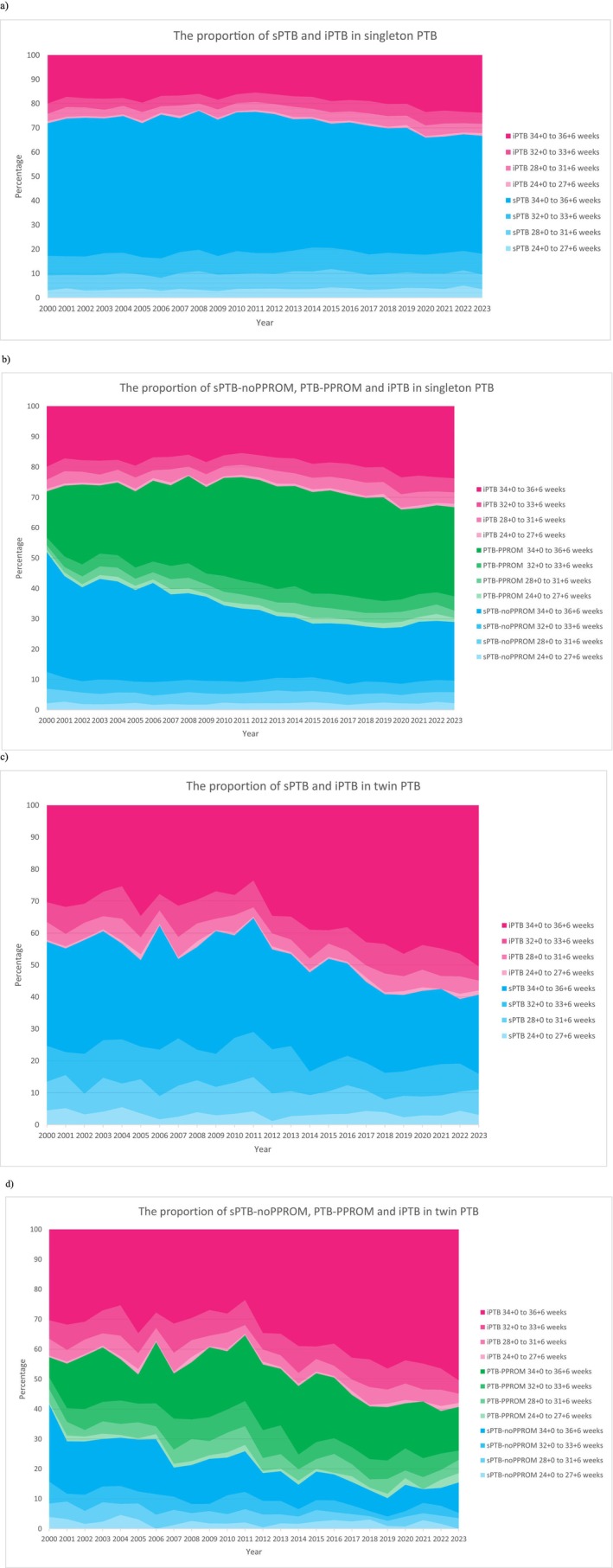
The proportion of different types of PTB (a) singleton—sPTB and iPTB, (b) singleton—sPTB‐noPPROM, PTB‐PPROM and iPTB, (c) twin—sPTB and iPTB, and (d) twin—sPTB‐noPPROM, PTB‐PPROM and iPTB.

Figure 2a shows the secular annual proportion of sPTB and iPTB in singletons across various gestations. sPTB contributed to a larger proportion of PTB, but its proportion reduced from 72.03% to 66.82%. A significant increase was noted in both overall sPTB (AAPC 0.44, 95% CI 0.11 to 0.76) and iPTB (AAPC 2.01, 95% CI 1.57 to 2.45) between 2000 and 2023 (Table 1). In addition, all subgroups showed notable increases in the PTB rates, except for late sPTB (Table S3).

### 3.3 sPTB‐noPPROM Versus PTB‐PPROM Versus iPTB—Singleton

Figure 2b shows the secular annual proportion of sPTB‐noPPROM, PTB‐PPROM, and iPTB in singletons across various gestations. PTB‐PPROM became the leading cause of PTB, and its proportion increased from 20.00% to 37.87%. PTB‐PPROM dramatically increased in both overall and its subgroups from 2000 to 2023. Furthermore, significant reductions were observed in both the overall and late sPTB‐noPPROM rates (Table 1 and Table S3).

### 3.4 Twin Pregnancy

The numbers and rates of PTB in twin pregnancies at different gestations between 2000 and 2023 are shown in Table 1, Table S5 and Figure 1b. The overall PTB rate increased significantly from 45.76% in 2000 to 62.43% in 2023 (AAPC 1.15, 95% CI 0.66 to 1.57). The rate of extreme and late PTB also increased significantly between 2000 and 2023 (Table S5). Supplementary analysis on the PTB rate as per total births (i.e., stillbirths included) yielded similar trends; the overall PTB rate increased significantly from 45.78% to 62.53% (AAPC 1.42, 95% CI 0.77 to 1.78) (Table S4).

### 3.5 sPTB Versus iPTB—Twin

Figure 2c shows the annual proportion of sPTB and iPTB. In 2000, sPTB and iPTB contributed to 57.30% and 42.70% of PTB. By 2023, the proportion of sPTB decreased to 40.71% while iPTB became the major contributor to PTB at 59.29%. The overall iPTB rate increased significantly (AAPC 2.75, 95% CI 1.82 to 3.69). Apart from an increase in the rate of late iPTB, there were no other notable changes observed amongst iPTB and sPTB in twins (Table 1 and Table S5).

### 3.6 sPTB‐noPPROM Versus PTB‐PPROM Versus iPTB—Twin

Figure 2d shows the annual proportion of sPTB‐noPPROM, PTB‐PPROM, and iPTB in twins across various gestations. iPTB remained the major contributor of PTB throughout the study period. The PTB‐PPROM rate increased significantly overall, moderate, and late PTB. On the other hand, the rate of sPTB‐noPPROM reduced significantly overall, moderate, and late PTB (Table 1 and Table S5).

### 3.7 Indication for iPTB

Table S6 shows the indication of iPTB amongst singleton and twin pregnancies. Figure S2 shows the proportion of each indication. Hypertensive disorders were the predominant underlying causes in both singleton and twin pregnancies, accounting for 44.47% and 31.97% of iPTB, respectively. In addition, it demonstrated an increasing trend from 2000 to 2023 amongst singleton pregnancies (AAPC 1.91, 95% CI 1.41 to 2.28).

**FIGURE 3:**
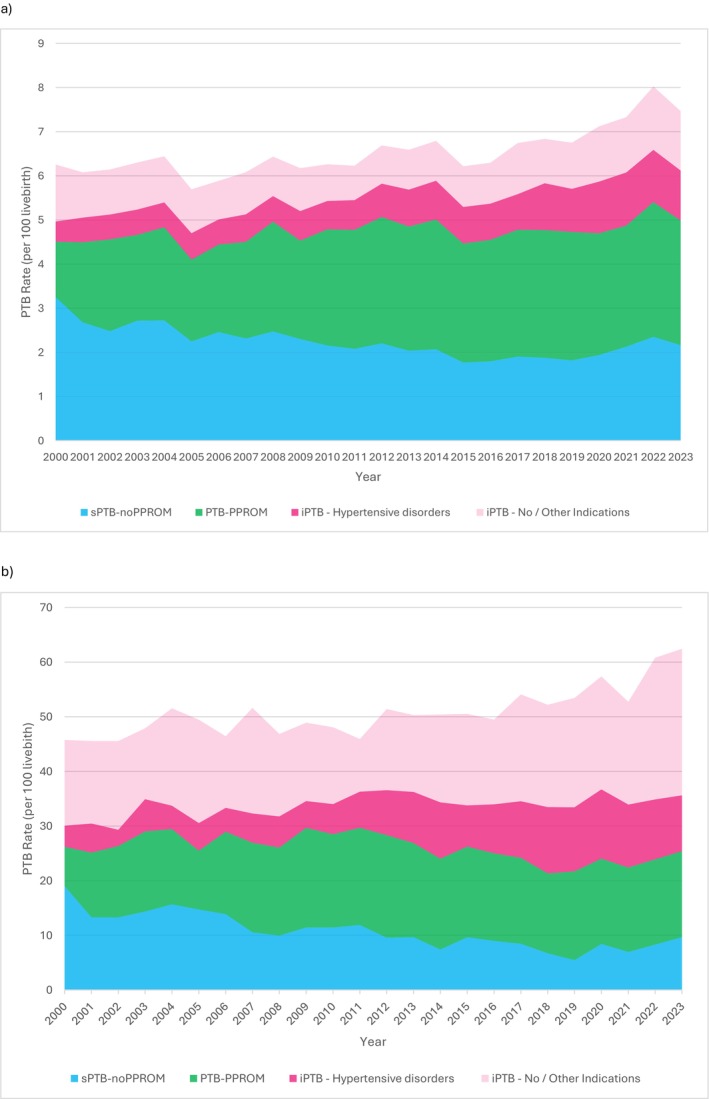
The contribution of each type of PTB to the overall rise in the PTB rate (a) singletons, (b) twins.

The trend of intrauterine growth restriction as a cause of iPTB for twin pregnancies increased during the study period. On the other hand, the proportion of iPTB as a result of maternal diseases in both singleton and twin pregnancies, antepartum haemorrhage in singleton pregnancies, and both abnormal cardiotocogram and twin pregnancy itself as indication for iPTB in twin pregnancies declined over the years. Figure 3 shows the component of sPTB‐noPPROM, PTB‐PPROM, iPTB for hypertensive disorders and iPTB for other indications with respect to the rising PTB rates.

### 3.8 Stillbirth, Perinatal and Neonatal Mortality

Table 2 and Table S7 show the numbers and rates of stillbirth and perinatal death amongst singleton and twin deliveries at < 37 weeks of gestation. Singleton pregnancies demonstrated no discernible patterns in the rates of stillbirth and perinatal mortality amongst overall and various PTB at different gestational ages between 2000 and 2023. In twin pregnancies, there were significant reductions in the rate of stillbirth amongst deliveries < 37 weeks (AAPC −4.58, 95% CI −7.48 to −1.62) and perinatal death < 37 weeks (AAPC −4.31, 95% CI −6.55 to −2.00).

**TABLE 2:** The number and rate (per 1000 total births) of stillbirth and perinatal death amongst singleton and twin deliveries at < 37 weeks of gestation.

Singleton pregnancy																												
	Total	2000	2001	2002	2003	2004	2005	2006	2007	2008	2009	2010	2011	2012	2013	2014	2015	2016	2017	2018	2019	2020	2021	2022	2023	% Change 2000–2023	Average annual percent change (95% CI)	*p*
	*n* = 56 171	*n* = 2486	*n* = 2218	*n* = 2317	*n* = 2269	*n* = 2427	*n* = 2379	*n* = 2377	*n* = 2405	*n* = 2091	*n* = 2533	*n* = 2682	*n* = 2856	*n* = 2974	*n* = 2430	*n* = 2701	*n* = 2458	*n* = 2549	*n* = 2549	*n* = 2405	*n* = 2289	*n* = 1915	*n* = 1735	*n* = 1579	*n* = 1547			
Stillbirth	1782 (31.72)	86 (34.59)	71 (32.01)	86 (37.12)	82 (36.14)	61 (25.13)	88 (36.99)	60 (25.24)	70 (29.11)	61 (29.17)	81 (31.98)	70 (26.10)	93 (32.56)	86 (28.92)	78 (32.10)	72 (26.66)	82 (33.36)	84 (32.95)	92 (36.09)	67 (27.86)	68 (29.71)	72 (37.60)	72 (41.50)	47 (29.77)	53 (34.26)	−0.97	0.24 (−0.84, 1.29)	0.656
Perinatal Death	2305 (41.04)	113 (45.45)	92 (41.48)	106 (45.75)	98 (43.19)	85 (35.02)	117 (49.18)	78 (32.81)	90 (37.42)	84 (40.17)	105 (41.45)	101 (37.66)	115 (40.27)	115 (38.67)	109 (44.86)	95 (35.17)	109 (44.34)	110 (43.15)	112 (43.94)	84 (34.93)	86 (37.57)	91 (47.52)	84 (48.41)	64 (40.53)	62 (40.08)	−11.83	0.03 (−0.85, 0.92)	0.957

Twin pregnancy																												
	Total	2000	2001	2002	2003	2004	2005	2006	2007	2008	2009	2010	2011	2012	2013	2014	2015	2016	2017	2018	2019	2020	2021	2022	2023	% Change 2000–2023	Average annual percent change (95% CI)	*p*
	*n* = 13 544	*n* = 358	*n* = 310	*n* = 374	*n* = 342	*n* = 438	*n* = 454	*n* = 468	*n* = 642	*n* = 464	*n* = 680	*n* = 706	*n* = 766	*n* = 844	*n* = 666	*n* = 736	*n* = 672	*n* = 764	*n* = 748	*n* = 714	*n* = 686	*n* = 544	*n* = 348	*n* = 366	*n* = 454			
Stillbirth	180 (13.29)	6 (16.76)	5 (16.13)	9 (24.06)	3 (8.77)	11 (25.11)	10 (22.03)	4 (8.55)	15 (23.36)	7 (15.09)	16 (23.53)	17 (24.08)	5 (6.53)	12 (14.22)	5 (7.51)	8 (10.87)	7 (10.42)	8 (10.47)	7 (9.36)	7 (9.80)	5 (7.29)	3 (5.51)	2 (5.75)	2 (5.46)	6 (13.22)	−21.15	−4.58 (−7.48, −1.62)	0.003
Perinatal death	287 (21.19)	15 (41.90)	8 (25.81)	11 (29.41)	10 (29.24)	16 (36.53)	16 (35.24)	8 (17.09)	21 (32.71)	11 (23.71)	22 (32.35)	20 (28.33)	16 (20.89)	14 (16.59)	5 (7.51)	11 (14.95)	10 (14.88)	13 (17.02)	15 (20.05)	10 (14.01)	10 (14.58)	5 (9.19)	7 (20.11)	5 (13.66)	8 (17.62)	−57.94	−4.31 (−6.55, −2.00)	< 0.001

Table 3, Tables S8 and S9 show the numbers and rates of neonatal death amongst singleton and twin deliveries at < 37 weeks of gestation. For singleton, overall very PTB (AAPC −3.31, 95% CI −5.79 to −0.76) and iPTB < 37 weeks (overall AAPC −3.46, 95% CI −5.27 to −1.27; moderate AAPC −58.47, 95% CI −80.13 to −14.46) showed noteworthy reducing trends in neonatal death. In twin pregnancy, the rate of neonatal death amongst overall PTB < 37 weeks (AAPC −3.07, 95% CI −5.97 to −0.07) and late iPTB (AAPC −5.16, 95% CI −9.32 to −1.05) reduced significantly from 2000 to 2023.

**TABLE 3:** The number and rate (per 1000 livebirth) of neonatal death amongst singleton and twin deliveries at < 37 weeks of gestation.

Singleton pregnancy																												
	Total	2000	2001	2002	2003	2004	2005	2006	2007	2008	2009	2010	2011	2012	2013	2014	2015	2016	2017	2018	2019	2020	2021	2022	2023	% Change 2000–2023	Average annual percent change (95% CI)	*p*
	*n* = 54 389	*n* = 2400	*n* = 2147	*n* = 2231	*n* = 2187	*n* = 2366	*n* = 2291	*n* = 2317	*n* = 2335	*n* = 2030	*n* = 2452	*n* = 2612	*n* = 2763	*n* = 2888	*n* = 2352	*n* = 2629	*n* = 2376	*n* = 2465	*n* = 2457	*n* = 2338	*n* = 2221	*n* = 1843	*n* = 1663	*n* = 1532	*n* = 1494			
Overall	667 (12.26)	32 (13.33)	28 (13.04)	22 (9.86)	22 (10.06)	30 (12.68)	36 (15.71)	23 (9.93)	27 (11.56)	34 (16.75)	27 (11.01)	43 (16.46)	28 (10.13)	38 (13.16)	40 (17.01)	30 (11.41)	32 (13.47)	28 (11.36)	32 (13.02)	24 (10.27)	23 (10.36)	23 (12.48)	15 (9.02)	20 (13.05)	10 (6.69)	−49.80	−0.89 (−2.45, 0.65)	0.247
sPTB‐noPPROM	261 (13.76)	13 (10.42)	16 (16.91)	10 (11.11)	9 (9.54)	12 (11.99)	16 (17.70)	7 (7.22)	11 (12.39)	11 (14.10)	13 (14.22)	18 (20.02)	12 (13.00)	14 (14.72)	15 (20.63)	14 (17.48)	9 (13.31)	10 (14.22)	13 (18.73)	9 (14.02)	10 (16.72)	4 (7.97)	2 (4.14)	9 (20.04)	4 (9.26)	−11.11	−0.28 (−2.87, 2.37)	0.805
PTB‐PPROM	171 (8.29)	4 (8.33)	3 (4.69)	2 (2.65)	3 (4.44)	8 (10.32)	10 (13.42)	8 (10.26)	6 (7.14)	14 (17.86)	5 (5.64)	13 (11.84)	7 (5.85)	10 (8.08)	14 (13.94)	9 (7.91)	13 (12.61)	7 (6.49)	8 (7.63)	5 (5.05)	5 (5.22)	6 (8.40)	3 (4.82)	6 (10.29)	2 (3.54)	−57.52	−0.35 (−3.90, 3.29)	0.829
iPTB	234 (15.84)	15 (22.35)	9 (16.10)	10 (17.42)	10 (17.61)	10 (16.95)	10 (15.63)	8 (14.08)	10 (16.53)	9 (19.35)	9 (13.80)	12 (19.54)	9 (13.98)	13 (18.60)	11 (17.71)	7 (10.16)	10 (14.95)	11 (16.11)	11 (15.38)	10 (14.16)	8 (12.03)	13 (20.73)	10 (17.95)	5 (10.00)	4 (8.08)	−63.85	−3.46 (−5.27, −1.27)	0.019

Twin pregnancy																												
	Total	2000	2001	2002	2003	2004	2005	2006	2007	2008	2009	2010	2011	2012	2013	2014	2015	2016	2017	2018	2019	2020	2021	2022	2023	% Change 2000–2023	Average annual percent change (95% CI)	*p*
	*n* = 13 364	*n* = 352	*n* = 305	*n* = 365	*n* = 339	*n* = 427	*n* = 444	*n* = 464	*n* = 627	*n* = 457	*n* = 664	*n* = 689	*n* = 761	*n* = 832	*n* = 661	*n* = 728	*n* = 665	*n* = 756	*n* = 741	*n* = 707	*n* = 681	*n* = 541	*n* = 346	*n* = 364	*n* = 448			
Overall	140 (10.48)	9 (25.57)	3 (9.84)	2 (5.48)	7 (20.65)	7 (16.39)	8 (18.02)	5 (10.78)	8 (12.76)	6 (13.13)	8 (12.05)	6 (8.71)	11 (14.45)	3 (3.61)	3 (4.54)	3 (4.12)	7 (10.53)	7 (9.26)	10 (13.50)	8 (11.32)	5 (7.34)	4 (7.39)	5 (14.45)	3 (8.24)	2 (4.46)	−82.54	−3.07 (−5.97, −0.07)	0.047
sPTB‐noPPROM	48 (17.45)	7 (48.28)	0 (0.00)	1 (9.35)	2 (19.61)	4 (31.25)	4 (30.08)	1 (7.19)	2 (15.63)	2 (20.62)	4 (25.97)	0 (0.00)	6 (30.46)	0 (0.00)	1 (7.87)	0 (0.00)	2 (15.63)	4 (29.41)	5 (43.10)	0 (0.00)	1 (14.49)	0 (0.00)	2 (44.44)	0 (0.00)	0 (0.00)	−100.00	−1.66 (−8.10, 5.01)	0.579
PTB‐PPROM	39 (9.35)	1 (17.86)	1 (12.50)	0 (0.00)	3 (29.13)	2 (17.39)	2 (20.83)	3 (19.74)	4 (20.20)	0 (0.00)	1 (3.98)	2 (8.20)	5 (17.01)	0 (0.00)	0 (0.00)	2 (8.26)	0 (0.00)	0 (0.00)	2 (9.26)	4 (20.00)	1 (4.81)	2 (13.70)	2 (19.61)	1 (10.64)	1 (8.77)	−50.88	3.88 (−45.79, 100.66)	0.922
iPTB	53 (8.23)	1 (6.62)	2 (14.71)	1 (6.58)	2 (14.93)	1 (5.43)	2 (9.30)	1 (5.78)	2 (6.64)	4 (19.80)	3 (11.58)	4 (14.23)	0 (0.00)	3 (8.04)	2 (6.54)	1 (2.62)	5 (15.72)	3 (8.00)	3 (7.33)	4 (9.55)	3 (7.43)	2 (6.35)	1 (5.03)	2 (9.05)	1 (3.77)	−43.02	−0.95 (−38.08, 58.77)	0.887

## 4 Discussion

### 4.1 Main Findings

We demonstrated significant increases in the PTB rates amongst singleton and twin pregnancy in a high‐income city. For singleton pregnancy, the rise was driven by the increases in both PTB‐PPROM and iPTB across extreme, very, moderate, and late preterm gestations. Similarly, for twin pregnancy, PTB‐PPROM and iPTB accounted for the increase in PTB. Further subclassification of sPTB into separate entities (sPTB‐noPPROM and PTB‐PPROM) has led to the observation of significant reductions in sPTB‐noPPROM (overall and late for singleton; overall, moderate, and late for twin pregnancy), along with significant upward trends in PTB‐PPROM (overall and all subgroups for singleton; overall, moderate, and late for twin pregnancy). Overtime, PTB‐PPROM became the dominant cause of singleton PTB while iPTB remained the most significant contributor for twin PTB. Hypertensive disorders were the main causes for iPTB, which increased over the years while maternal illnesses as indications for iPTB reduced. Reduction in the rate of neonatal mortality was noted amongst very PTB and overall iPTB for singleton, and iPTB for twin pregnancies between 34^+0^ and 36^+6^ weeks. Reduction in the stillbirth and perinatal mortality rates was also seen amongst PTB of twin pregnancies.

### 4.2 Interpretation

According to a recent systematic analysis, there was no observable change in the global estimation of PTB from 2010 to 2020. Out of 103 countries, only 27 countries achieved PTB reduction while the remaining 76 countries showed either an increased or a static PTB rate [1]. The lack of detailed evaluation on the classification of PTB, especially on how to classify PTB following PPROM, affected direct comparison with our data. Our data highlighted the importance of classifying PTB into different types. Knowing the trends and patterns of sPTB‐noPPROM, PTB‐PPROM, and iPTB are valuable for the exploration of reasons behind the temporal changes of PTB rates.

Spontaneous PTB rate could be influenced by the primary prevention of optimising population health, as well as the secondary prevention of utilising effective preventive strategies for women at higher risk of PTB, such as cerclage or progestogen for women with cervical insufficiency or a short cervix [11, 12]. Improving baseline health could also reduce iPTB since both spontaneous PTB and iPTB could share similar risk factors, as demonstrated by the decline of maternal illness as an indication for iPTB. In addition, the iPTB rate could be affected by changes in clinical practice influenced by guideline recommendations and prophylactic delivery attenuating pregnancy complications [13, 14, 15, 16]. Advancement in antenatal and neonatal care could sometimes paradoxically increase the PTB rate. Interventions could prolong the gestations amongst women who might otherwise miscarry. Enhancing surveillance for maternal and foetal conditions can lead to iPTB to avoid maternal complications and stillbirths [17]. Therefore, the temporal change in PTB is a continuous reflection of the dynamic interaction between the aetiologies of PTB (diseases) and the quality of healthcare provision. Global or national estimation of PTB often lack precise PTB subclassification [18]. The trend of PTB rate of different types should be interpreted in the context of the healthcare development and perinatal health indicators. One study documented improvement in neonatal survival amongst PTB for all gestational ages over the years [19]. Despite the escalating PTB rates, we also identified improving survival amongst singleton infants with iPTB as well as a reduction of stillbirth, perinatal and neonatal mortality rates for twin pregnancy with PTB. This may indicate improved identification of high‐risk pregnancies that may benefit from medically indicated PTB and better neonatal interventions.

The observed increase in iPTB amongst singleton and twin pregnancies in our cohort is consistent with the global phenomenon amongst high‐income settings [7, 20]. We found that iPTB due to hypertensive disorders had grown significantly over the years. In Hong Kong, an increasing number of women have chosen to delay childbearing in recent years [21]. As a result, the use of assisted reproductive techniques and advanced maternal age could lead to an increase in the incidence of gestational hypertensive disorders and thus hypertensive disorders related to iPTB [22, 23]. The reduction in spontaneous PTB in Hong Kong amongst singleton pregnancies was previously reported, which reflected the improvement in nutrition and environmental factors, as well as a better healthcare standard by providing prompt cerclage or progestogen treatment to women deemed at risk of PTB through screening in early pregnancy [4]. Unfortunately, implementing interventions could only achieve a relative reduction in PTB rates of 5% amongst high‐income countries [24]. A significant proportion of women with no apparent risk factors still delivered preterm [25, 26]. In addition, routine screening for short cervix by ultrasonography is not feasible in low to middle‐income countries where the burden of PTB remains high. On the other hand, PPROM also accounted for approximately 25%–40% of all PTB [7, 27, 28]. We demonstrated the effect of categorising PTB following PPROM as a distinct entity in our cohort, revealing that the apparent increase in sPTB amongst singletons was caused by the significant increase in PTB following PPROM, which emerged as the leading cause of PTB. There was a 126% and a 119% increase in the singleton and twin PTB rates following PPROM, which represented the largest relative increase in the PTB rate in our cohort. In Iceland, PTB following PPROM also increased from 1.3% to 1.7% between 1997 and 2016 [7]. The aetiology of PPROM is not fully understood. Potential contributors to PPROM are malnutrition, changing maternal demographics, ascending bacterial infection, cervical insufficiency, and invasive prenatal foetal interventions (such as amniocentesis, shunt insertions, fetoscopic laser ablation) [29]. A meta‐analysis of randomised controlled trials evaluated ten interventions involving supplementations, anti‐inflammatory agents, anti‐oxidant agents or antibiotics, which included the use of docosahexaenoic acid, aspirin, rofecoxib, vitamin C alone and with vitamin E, folic acid (alone, with iron, with iron and zinc, within micronutrient supplement), zinc, calcium, copper, and bacterial vaginosis treatment. Only folic acid with multiple micronutrient supplements reduced the risk of PPROM amongst an unselected cohort living in a low‐income setting. This finding needs to be affirmed in other settings before wider application. Therefore, the lack of proven prophylactic measures to prevent PPROM also accounted for the rising incidence [30]. We encourage researchers to focus on investigating preventive strategies for spontaneous PTB with or without PPROM, as well as alternatives that are practicable in resource‐limited areas.

### 4.3 Strengths and Limitations

Our data provided a robust investigation on the pattern and trend of different types of PTB across various gestations for singleton and twin pregnancies in Hong Kong. This study had limitations. First, PTB is a syndrome resulting from various aetiologies [31]. We did not classify PTB into different types based on maternal, placental or foetal pathology, which may help with understanding the genuine underlying pathologies leading to PTB and thus better prevention [32, 33, 34]. However, simpler categorisation to spontaneous, iatrogenic PTB and PTB following PPROM could allow for easier and more feasible implementation worldwide, especially in low resource settings where thorough investigations on the causes of PTB are often not possible. Second, data on interventions to prevent PTB (second trimester cervical length screening/use of progesterone or cerclage), antenatal treatments to optimise the outcomes of PTB (use of antenatal corticosteroid and magnesium sulphate), as well as maternal demographics were lacking, which prevented us from evaluating their potential effects on the trend of different types of PTB and from comparing the trends across regions. The observed trends in PTB may be influenced by temporal changes in maternal demographics within the population. For example, a higher proportion of pregnant women of advanced maternal age is associated with increased PTB rates. We would encourage reporting age‐standardised PTB trends when possible. Finally, data on delivery and neonatal death in private hospitals were not captured. Due to the limited neonatal support for preterm infants in the private setting, most private hospitals do not admit women with a potential risk of PTB, and admissions of preterm neonates are also expected to be minimal. So, our data may have overestimated the population PTB rate and neonatal death rate in Hong Kong.

## 5 Conclusions

In Hong Kong, PTB rates increased for singleton and twin pregnancies, but improved perinatal survival amongst infants born preterm was simultaneously observed. The rise in PTB was driven by PTB‐PPROM and iPTB, despite a decline in sPTB‐noPPROM. We encourage future studies to report PTB categorised into distinct types (spontaneous, iatrogenic PTB, and PTB following PPROM). Perinatal health indicators should be taken into consideration while interpreting the trajectory of the PTB rate. Preventive interventions are currently insufficient to reduce the burden of PTB, and there is an urgent need for research on strategies to prevent PTB of various types.

## Author Contributions

K.W.C. conceived the study and designed the protocol. K.W.C., T.S.‐T.A., T.O.C. analysed the data. K.W.C. wrote the first draft of the paper. K.W.C., T.S.‐T.A. and T.O.C. have verified the data. All authors critically revised the drafts of the paper. All authors had read, approved the final manuscript and were responsible for the decision to submit the manuscript.

## Funding

The authors have nothing to report.

## Disclosure

K.W.C. and T.S.‐T.A. are the guarantors of this work and, as such, had full access to all the data in the study and take responsibility for the integrity of the data and the accuracy of the data analysis.

## Conflicts of Interest

The authors declare no conflicts of interest.

## Supporting information


**Figure S1:** Flowchart showing the identification, inclusion and exclusion of study subjects.
**Table S1:** ICD‐9 diagnosis codes for each grouping of iPTB indications.
**Table S2:** Basic demographics of included subjects.
**Table S3:** The numbers and rates of preterm birth across various gestations amongst singleton pregnancies from 2000 to 2023.
**Table S4:** The numbers and rates of preterm birth per total births (livebirths and stillbirths) across various gestations from 2000 to 2023.
**Table S5:** The numbers and rates of preterm birth across various gestations amongst twin pregnancies from 2000 to 2023.
**Table S6:** Indication of all iPTB amongst singleton and twin pregnancies.
**Figure S2:** The proportion of different indications for iPTB in (a) singletons, (b) twins.
**Table S7:** The number and rate (per 1000 total births) of stillbirth and perinatal death amongst singleton and twin deliveries at < 37 weeks of gestation.
**Table S8:** The number and rate (per 1000 livebirth) of neonatal death amongst singleton deliveries at < 37 weeks of gestation.
**Table S9:** The number and rate (per 1000 livebirth) of neonatal death amongst twin deliveries at < 37 weeks of gestation.

## Data Availability

The datasets generated during and/or analysed in the current study are available from the corresponding author upon reasonable request.
