# Supplementary material for: Temporal Patterns in Preterm Birth Subtypes and Perinatal Survival, 2000–2023: Population‐Based, Repeated Cross‐Sectional Time‐Series
Source: BJOG. 2026 Jan 26;133(6):1213–26. doi: 10.1111/1471-0528.70166 (PMC13040435; doi:10.1111/1471-0528.70166)
Supplement: Supplementary file 1 — Figure S1: Flowchart showing the identification, inclusion and exclusion of study subjects. Table S1: ICD‐9 diagnosis codes for each grouping of iPTB indications. Table S2: Basic demographics of included subjects. Table S3: The numbers and rates of preterm birth across various gestations amongst singleton pregnancies from 2000 to 2023. Table S4: The numbers and rates of preterm birth per total births (livebirths and stillbirths) across various gestations from 2000 to 2023. Table S5: The numbers and rates of preterm birth across various gestations amongst twin pregnancies from 2000 to 2023. Table S6: Indication of all iPTB amongst singleton and twin pregnancies. Figure S2: The proportion of different indications for iPTB in (a) singletons, (b) twins. Table S7: The number and rate (per 1000 total births) of stillbirth and perinatal death amongst singleton and twin deliveries at < 37 weeks of gestation. Table S8: The number and rate (per 1000 livebirth) of neonatal death amongst singleton deliveries at < 37 weeks of gestation. Table S9: The number and rate (per 1000 livebirth) of neonatal death amongst twin deliveries at < 37 weeks of gestation. [file BJO-133-1213-s001.docx]

**Supplementary material - appendix**

Supplementary figure 1. Flowchart showing the identification, inclusion and exclusion of study subjects………...…………….. ……………………………...…………….. …………………2

Supplementary table 1. ICD-9 diagnosis codes for each grouping of iPTB indications……………..3

Supplementary table 2. Basic demographics of included subjects…………………………….4

Supplementary table 3. The numbers and rates of preterm birth across various gestations among singleton pregnancies from 2000 to 2023………...…………….. ……………………5

Supplementary table 4. The numbers and rates of preterm birth per total births (livebirths and stillbirths) across various gestations from 2000 to 2023………………………………………8

Supplementary table 5. The numbers and rates of preterm birth across various gestations among twin pregnancies from 2000 to 2023.………….………………..……………………..10

Supplementary table 6. Indication of all iPTB among singleton and twin pregnancies...…...14

Supplementary figure 2. The proportion of different indications for iPTB in a) singletons, b) twins………………………………………………………………………………………….16

Supplementary table 7. The number and rate (per 1000 total births) of stillbirth and perinatal death among singleton and twin deliveries at < 37 weeks of gestation………...……………18

Supplementary table 8. The number and rate (per 1000 livebirth) of neonatal death among singleton deliveries at < 37 weeks of gestation……………………………………………...20

Supplementary table 9. The number and rate (per 1000 livebirth) of neonatal death among twin deliveries at < 37 weeks of gestation…………………………………………………...22

Supplementary figure 1. Flowchart showing the identification, inclusion and exclusion of study subjects


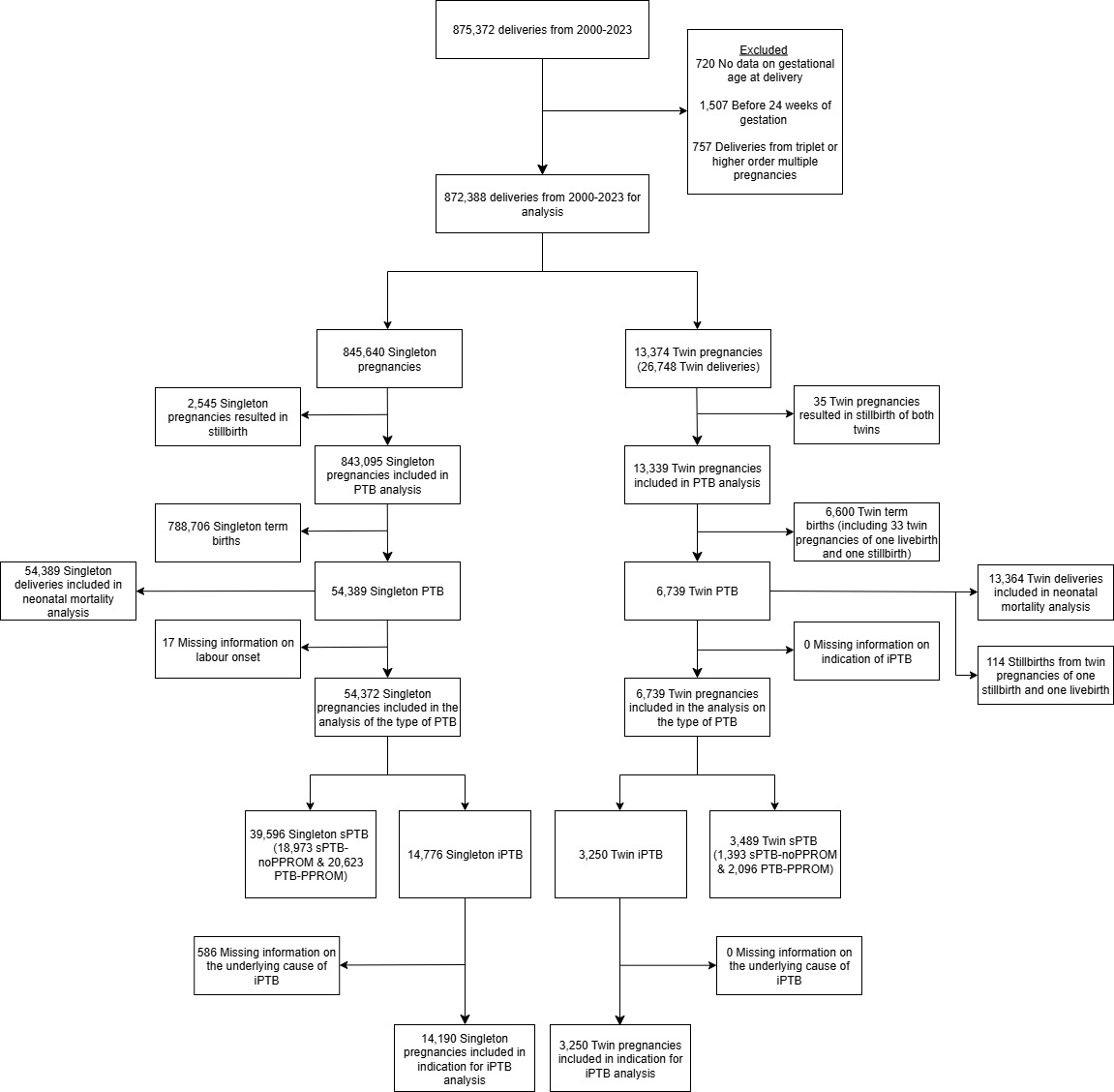


| Supplementary table 1. ICD-9 diagnosis codes for each grouping of iPTB indications | |
| --- | --- |
| Hypertensive Disorders | 401.0:0, 401.9:0, 401.9:3, 642.00:1, 642.01:801, 642.03:0, 642.03:800, 642.03:801, 642.21:801, 642.23:800, 642.30:2, 642.30:3, 642.30:4, 642.30:5, 642.31:801, 642.33:801, 642.33:803, 642.40:0, 642.40:1, 642.41:801, 642.43:0, 642.43:2, 642.43:800, 642.43:801, 642.44:0, 642.50:0, 642.51:0, 642.51:2, 642.51:4, 642.51:800, 642.52:0, 642.53:0, 642.53:2, 642.53:800, 642.60:0, 642.61:800, 642.63:0, 642.70:0, 642.70:1, 642.71:801, 642.73:800, 642.73:801, 642.90:0, 642.90:1, 642.91:0, 642.91:800, 642.93:0, 642.93:800 |
| Antepartum Haemorrhage | 641.00:0, 641.00:2, 641.00:3, 641.00:4, 641.00:5, 641.01:800, 641.03:0, 641.03:800, 641.03:802, 641.03:803, 641.03:804, 641.03:805, 641.10:0, 641.10:2, 641.10:3, 641.10:4, 641.10:5, 641.10:6, 641.10:7, 641.11:0, 641.11:1, 641.11:800, 641.13:800, 641.13:801, 641.13:802, 641.13:803, 641.13:804, 641.13:805, 641.20:0, 641.20:2, 641.20:3, 641.20:4, 641.21:0, 641.21:800, 641.21:801, 641.23:0, 641.23:800, 641.23:801, A641.90:0, 641.91:0, 641.91:801, 641.93:0, 641.93:800, 641.93:801 |
| Chorioamnionitis | 658.40:1 , 658.41:2, 658.43:800 |
| Abnormal Cardiotocogram | 656.30:0, 656.30:1, 656.30:3, 656.31:0, 656.31:800, 656.31:801, 656.33:0, 656.33:800, 656.33:801, 656.33:802, 656.90:1, 656.91:1, 656.91:2, 656.91:3, 656.93:1, 656.93:2, 656.93:801 |
| Twin Complications | 655.80:2, 655.81:801, 655.83:801 |
| Intrauterine Growth Restriction | 656.50:0, 656.50:1, 656.51:0, 656.51:801, 656.51:802, 656.53:0, 656.53:800, 656.53:801, 656.53:802, 655.80:10 |
| Oligohydramnios | 658.00:0, 658.00:1, 658.01:800, 658.03:0, 658.03:800 |
| Red Cell Isoimmunisation | 656.10:0, 656.13:800 |
| Cholestasis | 576.8:5, 576.8:6, 646.70:9, 646.70:10, 646.73:804 |
| Maternal Disease | 250.00:0, 250.00:2, 250.01:0, 250.13:0, 648.00:2, 648.00:3, 648.01:0, 648.01:1, 648.01:801, 648.01:802, 648.03:800, 648.80:0, 648.80:1, 648.80:2, 648.80:3, 648.80:4, 648.80:14, 648.81:2, 648.81:801, 648.81:803, 648.81:806, 648.81:807, 648.83:0, 648.83:34, 648.83:801, 648.90:2, 648.91:813, 648.93:813, 790.2:0 |
| Twin Pregnancy | 651:00:0, 651.00:1, 651.01:0, 651.03:800 |

| **Supplementary table 2. Basic demographics of included subjects** | | | |
| --- | --- | --- | --- |
|  | n (%) | | |
|  | Total  (n = 859014) | Singleton Pregnancy  (n = 845640) | Twin Pregnancy  (n = 13374) |
| **Maternal age** | | | |
| - < 20 | 12788 (1.49) | 12708 (1.50) | 80 (0.60) |
| - 20-24 | 92388 (10.76) | 91645 (10.84) | 743 (5.56) |
| - 25-29 | 231436 (26.94) | 229044 (27.09) | 2392 (17.89) |
| - 30-34 | 315307 (36.71) | 310117 (36.67) | 5190 (38.81) |
| - 35-39 | 173101 (20.15) | 168962 (19.98) | 4139 (30.95) |
| - 40-44 | 32376 (3.77) | 31665 (3.74) | 711 (5.32) |
| - ≥45 | 1614 (0.19) | 1495 (0.18) | 119 (0.89) |
| - Unknown | 4 (<0.01) | 4 (<0.01) | 0 (0.00) |
| **Ethnicity** | | | |
| - Chinese | 784656 (91.34) | 772316 (91.33) | 12340 (92.27) |
| - Other Asian | 44135 (5.14) | 43639 (5.16) | 496 (3.71) |
| - Non-Asian | 9301 (1.08) | 9003 (1.06) | 298 (2.23) |
| - Unknown | 20922 (2.44) | 20682 (2.45) | 240 (1.79) |
| **Parity** | | | |
| - Nulliparous | 443994 (51.69) | 435366 (51.48) | 8628 (64.51) |
| - Multiparous | 415020 (48.31) | 410274 (48.52) | 4746 (35.49) |
| **Stillbirth** | 2727 (0.32) | 2545 (0.30) | 182 (1.36)  Both twins: 35 (0.26)  Stillbirth of one twin: 147 (1.10) |

| **Supplementary table 3. The numbers and rates of preterm birth across various gestations among singleton pregnancies from 2000 to 2023.** | | | | | | | | | | | | | | | | | | | | | | | | | | | | |
| --- | --- | --- | --- | --- | --- | --- | --- | --- | --- | --- | --- | --- | --- | --- | --- | --- | --- | --- | --- | --- | --- | --- | --- | --- | --- | --- | --- | --- |
|  | **Total**  **(n = 843095)** | **2000 (n = 38362)** | **2001 (n = 35312)** | **2002 (n = 36315)** | **2003 (n = 34736)** | **2004 (n = 36754)** | **2005 (n = 40244)** | **2006 (n = 39383)** | **2007 (n = 38381)** | **2008 (n = 31542)** | **2009 (n = 39727)** | **2010 (n = 41721)** | **2011 (n = 44372)** | **2012 (n = 43181)** | **2013 (n = 35708)** | **2014 (n = 38713)** | **2015 (n = 38231)** | **2016 (n = 39160)** | **2017 (n = 36461)** | **2018 (n = 34209)** | **2019 (n = 32908)** | **2020 (n = 25885)** | **2021 (n = 22703)** | **2022 (n = 19097)** | **2023 (n = 19990)** | **% Change 2000-2023** | **Average annual percent change (95% CI)** | **p-value** |
| **Overall** | | | | | | | | | | | | | | | | | | | | | | | | | | | | |
| All preterm birth | 54389 (6.45) | 2400 (6.26) | 2147 (6.08) | 2231 (6.14) | 2187 (6.30) | 2366 (6.44) | 2291 (5.69) | 2317 (5.88) | 2335 (6.08) | 2030 (6.44) | 2452 (6.17) | 2612 (6.26) | 2763 (6.23) | 2888 (6.69) | 2352 (6.59) | 2629 (6.79) | 2376 (6.21) | 2465 (6.29) | 2457 (6.74) | 2338 (6.83) | 2221 (6.75) | 1843 (7.12) | 1663 (7.33) | 1532 (8.02) | 1494 (7.47) | 19.46 | 1.06 (0.59, 1.44) | < 0.001 |
| Extreme preterm birth | 2356 (0.28) | 91 (0.24) | 99 (0.28) | 80 (0.22) | 83 (0.24) | 99 (0.27) | 104 (0.26) | 85 (0.22) | 106 (0.28) | 74 (0.23) | 87 (0.22) | 108 (0.26) | 122 (0.27) | 133 (0.31) | 102 (0.29) | 113 (0.29) | 123 (0.32) | 115 (0.29) | 101 (0.28) | 97 (0.28) | 116 (0.35) | 85 (0.33) | 75 (0.33) | 88 (0.46) | 70 (0.35) | 47.62 | 1.98 (1.15, 2.79) | < 0.001 |
| Very preterm birth | 5480 (0.65) | 223 (0.58) | 204 (0.58) | 221 (0.61) | 200 (0.58) | 243 (0.66) | 217 (0.54) | 184 (0.47) | 254 (0.66) | 211 (0.67) | 249 (0.63) | 246 (0.59) | 269 (0.61) | 268 (0.62) | 274 (0.77) | 281 (0.73) | 269 (0.70) | 261 (0.67) | 256 (0.70) | 250 (0.73) | 229 (0.70) | 191 (0.74) | 179 (0.79) | 155 (0.81) | 146 (0.73) | 25.64 | 1.43 (0.82, 2.04) | < 0.001 |
| Moderate preterm birth | 6934 (0.82) | 295 (0.77) | 255 (0.72) | 257 (0.71) | 296 (0.85) | 272 (0.74) | 255 (0.63) | 284 (0.72) | 298 (0.78) | 258 (0.82) | 286 (0.72) | 343 (0.82) | 337 (0.76) | 365 (0.85) | 303 (0.85) | 387 (1.00) | 316 (0.83) | 333 (0.85) | 331 (0.91) | 321 (0.94) | 276 (0.84) | 245 (0.95) | 231 (1.02) | 194 (1.02) | 196 (0.98) | 27.50 | 1.48 (0.99, 1.97) | < 0.001 |
| Late preterm birth | 39619 (4.70) | 1791 (4.67) | 1589 (4.50) | 1673 (4.61) | 1608 (4.63) | 1752 (4.77) | 1715 (4.26) | 1764 (4.48) | 1677 (4.37) | 1487 (4.71) | 1830 (4.61) | 1915 (4.59) | 2035 (4.59) | 2122 (4.91) | 1673 (4.69) | 1848 (4.77) | 1668 (4.36) | 1756 (4.48) | 1769 (4.85) | 1670 (4.88) | 1600 (4.86) | 1322 (5.11) | 1178 (5.19) | 1095 (5.73) | 1082 (5.41) | 15.94 | 0.86 (0.48, 1.19) | < 0.001 |
| **sPTB** | | | | | | | | | | | | | | | | | | | | | | | | | | | | |
| All preterm birth | 39596 (4.70) | 1728 (4.51) | 1586 (4.49) | 1655 (4.56) | 1618 (4.66) | 1776 (4.83) | 1649 (4.10) | 1749 (4.44) | 1728 (4.50) | 1564 (4.96) | 1800 (4.53) | 1997 (4.79) | 2119 (4.78) | 2188 (5.07) | 1731 (4.85) | 1939 (5.01) | 1707 (4.47) | 1781 (4.55) | 1742 (4.78) | 1632 (4.77) | 1556 (4.73) | 1216 (4.70) | 1106 (4.87) | 1032 (5.41) | 997 (4.99) | 10.74 | 0.44 (0.11, 0.76) | 0.008 |
| Extreme preterm birth | 1936 (0.23) | 71 (0.19) | 84 (0.24) | 65 (0.18) | 68 (0.20) | 84 (0.23) | 85 (0.21) | 66 (0.17) | 84 (0.22) | 66 (0.21) | 68 (0.17) | 96 (0.23) | 102 (0.23) | 109 (0.25) | 81 (0.23) | 94 (0.24) | 102 (0.27) | 97 (0.25) | 78 (0.21) | 82 (0.24) | 92 (0.28) | 73 (0.28) | 60 (0.26) | 76 (0.40) | 53 (0.27) | 43.27 | 1.96 (1.18, 2.76) | < 0.001 |
| Very preterm birth | 3506 (0.42) | 150 (0.39) | 117 (0.33) | 142 (0.39) | 137 (0.39) | 160 (0.44) | 131 (0.33) | 124 (0.31) | 154 (0.40) | 157 (0.50) | 165 (0.42) | 159 (0.38) | 178 (0.40) | 173 (0.40) | 175 (0.49) | 191 (0.49) | 179 (0.47) | 168 (0.43) | 154 (0.42) | 147 (0.43) | 142 (0.43) | 110 (0.43) | 105 (0.46) | 97 (0.51) | 91 (0.46) | 16.44 | 1.11 (0.41, 1.81) | 0.001 |
| Moderate preterm birth | 4564 (0.54) | 193 (0.50) | 166 (0.47) | 173 (0.48) | 197 (0.57) | 196 (0.53) | 166 (0.41) | 185 (0.47) | 202 (0.53) | 179 (0.57) | 188 (0.47) | 247 (0.59) | 229 (0.52) | 249 (0.58) | 201 (0.56) | 258 (0.67) | 207 (0.54) | 217 (0.55) | 206 (0.57) | 204 (0.60) | 167 (0.51) | 143 (0.55) | 142 (0.63) | 122 (0.64) | 127 (0.64) | 26.29 | 1.07 (0.50, 1.64) | < 0.001 |
| Late preterm birth | 29590 (3.51) | 1314 (3.43) | 1219 (3.45) | 1275 (3.51) | 1216 (3.50) | 1336 (3.64) | 1267 (3.15) | 1374 (3.49) | 1288 (3.36) | 1162 (3.68) | 1379 (3.47) | 1495 (3.58) | 1610 (3.63) | 1657 (3.84) | 1274 (3.57) | 1396 (3.61) | 1219 (3.19) | 1299 (3.32) | 1304 (3.58) | 1199 (3.51) | 1155 (3.51) | 890 (3.44) | 799 (3.52) | 737 (3.86) | 726 (3.63) | 6.04 | 0.16 (-0.13, 0.45) | 0.285 |
| **sPTB-noPPROM** | | | | | | | | | | | | | | | | | | | | | | | | | | | | |
| All preterm birth | 18973 (2.25) | 1248 (3.25) | 946 (2.68) | 900 (2.48) | 943 (2.72) | 1001 (2.72) | 904 (2.25) | 969 (2.46) | 888 (2.31) | 780 (2.47) | 914 (2.30) | 899 (2.16) | 923 (2.08) | 951 (2.20) | 727 (2.04) | 801 (2.07) | 676 (1.77) | 703 (1.80) | 694 (1.90) | 642 (1.88) | 598 (1.82) | 502 (1.94) | 483 (2.13) | 449 (2.35) | 432 (2.16) | -33.56 | -0.96 (-1.67, -0.41) | <0.001 |
| Extreme preterm birth | 1157 (0.14) | 52 (0.14) | 58 (0.16) | 43 (0.12) | 40 (0.12) | 47 (0.13) | 53 (0.13) | 39 (0.10) | 45 (0.12) | 35 (0.11) | 43 (0.11) | 63 (0.15) | 59 (0.13) | 64 (0.15) | 51 (0.14) | 60 (0.16) | 60 (0.16) | 54 (0.14) | 40 (0.11) | 49 (0.14) | 54 (0.16) | 40 (0.15) | 34 (0.15) | 41 (0.21) | 33 (0.17) | 21.80 | 0.84 (-0.32, 2.33) | 0.179 |
| Very preterm birth | 1926 (0.23) | 115 (0.30) | 79 (0.22) | 84 (0.23) | 75 (0.22) | 89 (0.24) | 78 (0.19) | 70 (0.18) | 77 (0.20) | 84 (0.27) | 90 (0.23) | 74 (0.18) | 93 (0.21) | 105 (0.24) | 100 (0.28) | 99 (0.26) | 89 (0.23) | 89 (0.23) | 81 (0.22) | 76 (0.22) | 67 (0.20) | 50 (0.19) | 59 (0.26) | 49 (0.26) | 54 (0.27) | -9.88 | 0.20 (-0.88, 1.26) | 0.707 |
| Moderate preterm birth | 2235 (0.27) | 133 (0.35) | 91 (0.26) | 84 (0.23) | 104 (0.30) | 98 (0.27) | 82 (0.20) | 103 (0.26) | 99 (0.26) | 82 (0.26) | 101 (0.25) | 111 (0.27) | 103 (0.23) | 114 (0.26) | 97 (0.27) | 115 (0.30) | 106 (0.28) | 99 (0.25) | 88 (0.24) | 94 (0.27) | 82 (0.25) | 68 (0.26) | 63 (0.28) | 61 (0.32) | 57 (0.29) | -17.75 | -0.70 (-1.39, 0.49) | 0.234 |
| Late preterm birth | 13655 (1.62) | 948 (2.47) | 718 (2.03) | 689 (1.90) | 724 (2.09) | 767 (2.09) | 691 (1.72) | 757 (1.92) | 667 (1.74) | 579 (1.84) | 680 (1.71) | 651 (1.56) | 668 (1.51) | 668 (1.55) | 479 (1.34) | 527 (1.36) | 421 (1.10) | 461 (1.18) | 485 (1.33) | 423 (1.24) | 395 (1.20) | 344 (1.33) | 327 (1.44) | 298 (1.56) | 288 (1.44) | -41.69 | -1.91 (-2.59, -1.29) | < 0.001 |
| **PTB-PPROM** | | | | | | | | | | | | | | | | | | | | | | | | | | | | |
| All preterm birth | 20623 (2.45) | 480 (1.25) | 640 (1.81) | 755 (2.08) | 675 (1.94) | 775 (2.11) | 745 (1.85) | 780 (1.98) | 840 (2.19) | 784 (2.49) | 886 (2.23) | 1098 (2.63) | 1196 (2.70) | 1237 (2.87) | 1004 (2.81) | 1138 (2.94) | 1031 (2.70) | 1078 (2.75) | 1048 (2.88) | 990 (2.89) | 958 (2.91) | 714 (2.76) | 623 (2.74) | 583 (3.05) | 565 (2.83) | 125.92 | 3.46 (2.89, 4.18) | < 0.001 |
| Extreme preterm birth | 779 (0.09) | 19 (0.05) | 26 (0.07) | 22 (0.06) | 28 (0.08) | 37 (0.10) | 32 (0.08) | 27 (0.07) | 39 (0.10) | 31 (0.10) | 25 (0.06) | 33 (0.08) | 43 (0.10) | 45 (0.10) | 30 (0.08) | 34 (0.09) | 42 (0.11) | 43 (0.11) | 38 (0.10) | 33 (0.10) | 38 (0.12) | 33 (0.13) | 26 (0.11) | 35 (0.18) | 20 (0.10) | 102.03 | 2.99 (1.54, 4.40) | < 0.001 |
| Very preterm birth | 1580 (0.19) | 35 (0.09) | 38 (0.11) | 58 (0.16) | 62 (0.18) | 71 (0.19) | 53 (0.13) | 54 (0.14) | 77 (0.20) | 73 (0.23) | 75 (0.19) | 85 (0.20) | 85 (0.19) | 68 (0.16) | 75 (0.21) | 92 (0.24) | 90 (0.24) | 79 (0.20) | 73 (0.20) | 71 (0.21) | 75 (0.23) | 60 (0.23) | 46 (0.20) | 48 (0.25) | 37 (0.19) | 102.90 | 4.35 (3.02, 5.77) | < 0.001 |
| Moderate preterm birth | 2329 (0.28) | 60 (0.16) | 75 (0.21) | 89 (0.25) | 93 (0.27) | 98 (0.27) | 84 (0.21) | 82 (0.21) | 103 (0.27) | 97 (0.31) | 87 (0.22) | 136 (0.33) | 126 (0.28) | 135 (0.31) | 104 (0.29) | 143 (0.37) | 101 (0.26) | 118 (0.30) | 118 (0.32) | 110 (0.32) | 85 (0.26) | 75 (0.29) | 79 (0.35) | 61 (0.32) | 70 (0.35) | 123.92 | 3.21 (1.69, 4.31) | < 0.001 |
| Late preterm birth | 15935 (1.89) | 366 (0.95) | 501 (1.42) | 586 (1.61) | 492 (1.42) | 569 (1.55) | 576 (1.43) | 617 (1.57) | 621 (1.62) | 583 (1.85) | 699 (1.76) | 844 (2.02) | 942 (2.12) | 989 (2.29) | 795 (2.23) | 869 (2.25) | 798 (2.09) | 838 (2.14) | 819 (2.25) | 776 (2.27) | 760 (2.31) | 546 (2.11) | 472 (2.08) | 439 (2.30) | 438 (2.19) | 129.68 | 3.42 (2.97, 3.91) | < 0.001 |
| **iPTB** | | | | | | | | | | | | | | | | | | | | | | | | | | | | |
| All preterm birth | 14776 (1.75) | 671 (1.75) | 559 (1.58) | 574 (1.58) | 568 (1.64) | 590 (1.61) | 640 (1.59) | 568 (1.44) | 605 (1.58) | 465 (1.47) | 652 (1.64) | 614 (1.47) | 644 (1.45) | 699 (1.62) | 621 (1.74) | 689 (1.78) | 669 (1.75) | 683 (1.74) | 715 (1.96) | 706 (2.06) | 665 (2.02) | 627 (2.42) | 557 (2.45) | 500 (2.62) | 495 (2.48) | 41.59 | 2.01 (1.57, 2.45) | < 0.001 |
| Extreme preterm birth | 420 (0.05) | 20 (0.05) | 15 (0.04) | 15 (0.04) | 15 (0.04) | 15 (0.04) | 19 (0.05) | 19 (0.05) | 22 (0.06) | 8 (0.03) | 19 (0.05) | 12 (0.03) | 20 (0.05) | 24 (0.06) | 21 (0.06) | 19 (0.05) | 21 (0.05) | 18 (0.05) | 23 (0.06) | 15 (0.04) | 24 (0.07) | 12 (0.05) | 15 (0.07) | 12 (0.06) | 17 (0.09) | 63.14 | 2.03 (0.53, 3.56) | 0.010 |
| Very preterm birth | 1973 (0.23) | 73 (0.19) | 87 (0.25) | 79 (0.22) | 63 (0.18) | 83 (0.23) | 85 (0.21) | 60 (0.15) | 100 (0.26) | 54 (0.17) | 84 (0.21) | 87 (0.21) | 91 (0.21) | 95 (0.22) | 99 (0.28) | 90 (0.23) | 90 (0.24) | 93 (0.24) | 102 (0.28) | 103 (0.30) | 87 (0.26) | 81 (0.31) | 74 (0.33) | 58 (0.30) | 55 (0.28) | 44.60 | 2.03 (1.06 2.99) | < 0.001 |
| Moderate preterm birth | 2368 (0.28) | 102 (0.27) | 89 (0.25) | 84 (0.23) | 99 (0.29) | 76 (0.21) | 89 (0.22) | 99 (0.25) | 95 (0.25) | 79 (0.25) | 98 (0.25) | 95 (0.23) | 108 (0.24) | 116 (0.27) | 102 (0.29) | 129 (0.33) | 109 (0.29) | 116 (0.30) | 125 (0.34) | 117 (0.34) | 109 (0.33) | 102 (0.39) | 89 (0.39) | 72 (0.38) | 69 (0.35) | 29.83 | 1.67 (0.91, 2.72) | < 0.001 |
| Late preterm birth | 10015 (1.19) | 476 (1.24) | 368 (1.04) | 396 (1.09) | 391 (1.13) | 416 (1.13) | 447 (1.11) | 390 (0.99) | 388 (1.01) | 324 (1.03) | 451 (1.14) | 420 (1.01) | 425 (0.96) | 464 (1.07) | 399 (1.12) | 451 (1.17) | 449 (1.17) | 456 (1.16) | 465 (1.28) | 471 (1.38) | 445 (1.35) | 432 (1.67) | 379 (1.67) | 358 (1.88) | 354 (1.77) | 42.74 | 1.97 (1.50, 2.44) | < 0.001 |

| **Supplementary table 4. The numbers and rates of preterm birth per total births (livebirths and stillbirths) across various gestations from 2000 to 2023.** | | | | | | | | | | | | | | | | | | | | | | | | | | | | |
| --- | --- | --- | --- | --- | --- | --- | --- | --- | --- | --- | --- | --- | --- | --- | --- | --- | --- | --- | --- | --- | --- | --- | --- | --- | --- | --- | --- | --- |
|  | **Total** | **2000** | **2001** | **2002** | **2003** | **2004** | **2005** | **2006** | **2007** | **2008** | **2009** | **2010** | **2011** | **2012** | **2013** | **2014** | **2015** | **2016** | **2017** | **2018** | **2019** | **2020** | **2021** | **2022** | **2023** | **% Change 2000-2023** | **Average annual percent change (95% CI)** | **p-value** |
| **Singleton pregnancy** | | | | | | | | | | | | | | | | | | | | | | | | | | | | |
|  | n = 845640 | n = 38492 | n = 35412 | n = 36443 | n = 34857 | n = 36847 | n = 40372 | n = 39488 | n = 38495 | n = 31635 | n = 39834 | n = 41824 | n = 44500 | n = 43295 | n = 35813 | n = 38820 | n = 38346 | n = 39281 | n = 36581 | n = 34300 | n = 33005 | n = 25982 | n = 22794 | n = 19162 | n = 20062 |  | | |
| All preterm birth | 56171 (6.64) | 2486 (6.46) | 2218 (6.26) | 2317 (6.36) | 2269 (6.51) | 2427 (6.59) | 2379 (5.89) | 2377 (6.02) | 2405 (6.25) | 2091 (6.61) | 2533 (6.36) | 2682 (6.41) | 2856 (6.42) | 2974 (6.87) | 2430 (6.79) | 2701 (6.96) | 2458 (6.41) | 2549 (6.49) | 2549 (6.97) | 2405 (7.01) | 2289 (6.94) | 1915 (7.37) | 1735 (7.61) | 1579 (8.24) | 1547 (7.71) | 19.39 | 1.08 (0.61, 1.44) | < 0.001 |
| Extreme preterm birth | 2934 (0.35) | 129 (0.34) | 123 (0.35) | 108 (0.30) | 111 (0.32) | 122 (0.33) | 127 (0.31) | 103 (0.26) | 132 (0.34) | 93 (0.29) | 116 (0.29) | 131 (0.31) | 151 (0.34) | 157 (0.36) | 125 (0.35) | 134 (0.35) | 149 (0.39) | 138 (0.35) | 136 (0.37) | 112 (0.33) | 134 (0.41) | 108 (0.42) | 109 (0.48) | 101 (0.53) | 85 (0.42) | 26.42 | 1.24 (0.42, 2.32) | 0.003 |
| Very preterm birth | 5999 (0.71) | 250 (0.65) | 223 (0.63) | 250 (0.69) | 220 (0.63) | 259 (0.70) | 242 (0.60) | 200 (0.51) | 270 (0.70) | 226 (0.71) | 273 (0.69) | 262 (0.63) | 298 (0.67) | 292 (0.67) | 299 (0.83) | 303 (0.78) | 295 (0.77) | 281 (0.72) | 284 (0.78) | 273 (0.80) | 253 (0.77) | 212 (0.82) | 202 (0.89) | 170 (0.89) | 162 (0.81) | 24.33 | 1.46 (0.86, 2.07) | < 0.001 |
| Moderate preterm birth | 7203 (0.85) | 302 (0.78) | 268 (0.76) | 262 (0.72) | 308 (0.88) | 279 (0.76) | 274 (0.68) | 293 (0.74) | 302 (0.78) | 268 (0.85) | 296 (0.74) | 358 (0.86) | 354 (0.80) | 379 (0.88) | 314 (0.88) | 401 (1.03) | 325 (0.85) | 354 (0.90) | 343 (0.94) | 331 (0.97) | 285 (0.86) | 258 (0.99) | 238 (1.04) | 205 (1.07) | 206 (1.03) | 30.87 | 1.52 (1.06, 2.00) | < 0.001 |
| Late preterm birth | 40035 (4.73) | 1805 (4.69) | 1604 (4.53) | 1697 (4.66) | 1630 (4.68) | 1767 (4.80) | 1736 (4.30) | 1781 (4.51) | 1701 (4.42) | 1504 (4.75) | 1848 (4.64) | 1931 (4.62) | 2053 (4.61) | 2146 (4.96) | 1692 (4.72) | 1863 (4.80) | 1689 (4.40) | 1776 (4.52) | 1786 (4.88) | 1689 (4.92) | 1617 (4.90) | 1337 (5.15) | 1186 (5.20) | 1103 (5.76) | 1094 (5.45) | 16.29 | 0.85 (0.47, 1.17) | < 0.001 |
| **Twin pregnancy** | | | | | | | | | | | | | | | | | | | | | | | | | | | | |
|  | n = 13374 | n = 391 | n = 339 | n = 408 | n = 356 | n = 423 | n = 458 | n = 505 | n = 619 | n = 493 | n = 693 | n = 730 | n = 833 | n = 818 | n = 662 | n = 730 | n = 664 | n = 770 | n = 689 | n = 684 | n = 641 | n = 474 | n = 330 | n = 301 | n = 363 |  | | |
| All preterm birth | 6772 (50.64) | 179 (45.78) | 155 (45.72) | 187 (45.83) | 171 (48.03) | 219 (51.77) | 227 (49.56) | 234 (46.34) | 321 (51.86) | 232 (47.06) | 340 (49.06) | 353 (48.36) | 383 (45.98) | 422 (51.59) | 333 (50.30) | 368 (50.41) | 336 (50.60) | 382 (49.61) | 374 (54.28) | 357 (52.19) | 343 (53.51) | 272 (57.38) | 174 (52.73) | 183 (60.80) | 227 (62.53) | 36.60 | 1.42 (0.77, 1.78) | < 0.001 |
| Extreme preterm birth | 293 (2.19) | 9 (2.30) | 10 (2.95) | 7 (1.72) | 9 (2.53) | 15 (3.55) | 13 (2.84) | 5 (0.99) | 11 (1.78) | 14 (2.84) | 13 (1.88) | 17 (2.33) | 18 (2.16) | 10 (1.22) | 11 (1.66) | 15 (2.05) | 13 (1.96) | 15 (1.95) | 22 (3.19) | 16 (2.34) | 13 (2.03) | 11 (2.32) | 5 (1.52) | 11 (3.65) | 10 (2.75) | 19.68 | 0.17 (-2.05, 2.42) | 0.907 |
| Very preterm birth | 852 (6.37) | 27 (6.91) | 22 (6.49) | 22 (5.39) | 25 (7.02) | 31 (7.33) | 36 (7.86) | 27 (5.35) | 53 (8.56) | 36 (7.30) | 42 (6.06) | 54 (7.40) | 52 (6.24) | 54 (6.60) | 39 (5.89) | 36 (4.93) | 39 (5.87) | 48 (6.23) | 40 (5.81) | 35 (5.12) | 39 (6.08) | 31 (6.54) | 18 (5.45) | 21 (6.98) | 25 (6.89) | -0.27 | -0.57 (-1.51, 0.37) | 0.224 |
| Moderate preterm birth | 1219 (9.11) | 31 (7.93) | 24 (7.08) | 35 (8.58) | 33 (9.27) | 53 (12.53) | 39 (8.52) | 46 (9.11) | 78 (12.60) | 39 (7.91) | 64 (9.24) | 71 (9.73) | 86 (10.32) | 81 (9.90) | 71 (10.73) | 60 (8.22) | 44 (6.63) | 64 (8.31) | 59 (8.56) | 63 (9.21) | 50 (7.80) | 46 (9.70) | 32 (9.70) | 29 (9.63) | 21 (5.79) | -27.03 | -0.42 (-1.65, 0.83) | 0.478) |
| Late preterm birth | 4408 (32.96) | 112 (28.64) | 99 (29.20) | 123 (30.15) | 104 (29.21) | 120 (28.37) | 139 (30.35) | 156 (30.89) | 179 (28.92) | 143 (29.01) | 221 (31.89) | 211 (28.90) | 227 (27.25) | 277 (33.86) | 212 (32.02) | 257 (35.21) | 240 (36.14) | 255 (33.12) | 253 (36.72) | 243 (35.53) | 241 (37.60) | 184 (38.82) | 119 (36.06) | 122 (40.53) | 171 (47.11) | 64.46 | 1.66 (1.13, 2.22) | < 0.001 |

| **Supplementary table 5. The numbers and rates of preterm birth across various gestations among twin pregnancies from 2000 to 2023.** | | | | | | | | | | | | | | | | | | | | | | | | | | | | |
| --- | --- | --- | --- | --- | --- | --- | --- | --- | --- | --- | --- | --- | --- | --- | --- | --- | --- | --- | --- | --- | --- | --- | --- | --- | --- | --- | --- | --- |
|  | **Total (n = 13339)** | **2000 (n = 389)** | **2001 (n = 338)** | **2002 (n = 406)** | **2003 (n = 355)** | **2004 (n = 421)** | **2005 (n = 456)** | **2006 (n = 505)** | **2007 (n = 616)** | **2008 (n = 491)** | **2009 (n = 691)** | **2010 (n = 726)** | **2011 (n = 832)** | **2012 (n = 815)** | **2013 (n = 662)** | **2014 (n = 728)** | **2015 (n = 663)** | **2016 (n = 768)** | **2017 (n = 686)** | **2018 (n = 684)** | **2019 (n = 640)** | **2020 (n = 474)** | **2021 (n = 330)** | **2022 (n = 301)** | **2023 (n = 362)** | **% Change 2000-2023** | **Average annual percent change (95% CI)** | **p-value** |
| **Overall** | | | | | | | | | | | | | | | | | | | | | | | | | | | | |
| All preterm birth | 6739 (50.52) | 178 (45.76) | 154 (45.56) | 185 (45.57) | 170 (47.89) | 217 (51.54) | 225 (49.34) | 234 (46.34) | 318 (51.62) | 230 (46.84) | 338 (48.91) | 349 (48.07) | 382 (45.91) | 419 (51.41) | 333 (50.30) | 367 (50.41) | 335 (50.53) | 380 (49.48) | 371 (54.08) | 357 (52.19) | 342 (53.44) | 272 (57.38) | 174 (52.73) | 183 (60.80) | 226 (62.43) | 36.44 | 1.15 (0.66, 1.57) | < 0.001 |
| Extreme preterm birth | 279 (2.09) | 9 (2.31) | 9 (2.66) | 7 (1.72) | 8 (2.25) | 14 (3.33) | 12 (2.63) | 5 (0.99) | 10 (1.62) | 13 (2.65) | 11 (1.59) | 15 (2.07) | 18 (2.16) | 9 (1.10) | 11 (1.66) | 14 (1.92) | 13 (1.96) | 15 (1.95) | 21 (3.06) | 16 (2.34) | 12 (1.88) | 11 (2.32) | 5 (1.52) | 11 (3.65) | 10 (2.76) | 19.40 | 1.99 (1.54, 2.45) | < 0.001 |
| Very preterm birth | 842 (6.31) | 26 (6.68) | 22 (6.51) | 21 (5.17) | 25 (7.04) | 31 (7.36) | 36 (7.89) | 27 (5.35) | 51 (8.28) | 36 (7.33) | 42 (6.08) | 53 (7.30) | 52 (6.25) | 53 (6.50) | 39 (5.89) | 36 (4.95) | 38 (5.73) | 47 (6.12) | 38 (5.54) | 35 (5.12) | 39 (6.09) | 31 (6.54) | 18 (5.45) | 21 (6.98) | 25 (6.91) | 3.33 | -0.82 (-1.93, 0.24) | 0.133 |
| Moderate preterm birth | 1215 (9.11) | 31 (7.97) | 24 (7.10) | 34 (8.37) | 33 (9.30) | 52 (12.35) | 38 (8.33) | 46 (9.11) | 78 (12.66) | 39 (7.94) | 64 (9.26) | 71 (9.78) | 86 (10.34) | 81 (9.94) | 71 (10.73) | 60 (8.24) | 44 (6.64) | 63 (8.20) | 59 (8.60) | 63 (9.21) | 50 (7.81) | 46 (9.70) | 32 (9.70) | 29 (9.63) | 21 (5.80) | -27.21 | -0.65 (-2.13, 0.82) | 0.359 |
| Late preterm birth | 4403 (33.01) | 112 (28.79) | 99 (29.29) | 123 (30.30) | 104 (29.30) | 120 (28.50) | 139 (30.48) | 156 (30.89) | 179 (29.06) | 142 (28.92) | 221 (31.98) | 210 (28.93) | 226 (27.16) | 276 (33.87) | 212 (32.02) | 257 (35.30) | 240 (36.20) | 255 (33.20) | 253 (36.88) | 243 (35.53) | 241 (37.66) | 184 (38.82) | 119 (36.06) | 122 (40.53) | 170 (46.96) | 63.11 | 1.68 (1.06, 2.37) | < 0.001 |
| **sPTB** | | | | | | | | | | | | | | | | | | | | | | | | | | | | |
| All preterm birth | 3489 (26.16) | 102 (26.22) | 85 (25.15) | 107 (26.35) | 103 (29.01) | 124 (29.45) | 116 (25.49) | 146 (28.97) | 166 (26.95) | 128 (26.07) | 205 (29.67) | 207 (28.51) | 247 (29.69) | 231 (28.34) | 178 (26.89) | 175 (24.04) | 174 (26.24) | 192 (25.00) | 166 (24.20) | 146 (21.35) | 139 (21.72) | 114 (24.05) | 74 (22.42) | 72 (23.92) | 92 (25.41) | -3.08 | -0.20 (-0.95, 0.39) | 0.504 |
| Extreme preterm birth | 225 (1.69) | 8 (2.06) | 8 (2.37) | 6 (1.48) | 7 (1.97) | 13 (3.09) | 8 (1.76) | 4 (0.79) | 8 (1.30) | 9 (1.83) | 10 (1.45) | 12 (1.65) | 16 (1.92) | 6 (0.74) | 9 (1.36) | 11 (1.51) | 11 (1.66) | 13 (1.69) | 16 (2.33) | 14 (2.05) | 8 (1.25) | 8 (1.69) | 5 (1.52) | 8 (2.66) | 7 (1.93) | -5.97 | -0.02 (-2.16, 2.12) | 0.944 |
| Very preterm birth | 539 (4.04) | 16 (4.11) | 16 (4.73) | 12 (2.96) | 18 (5.07) | 16 (3.80) | 24 (5.27) | 17 (3.37) | 32 (5.19) | 23 (4.68) | 30 (4.34) | 34 (4.68) | 41 (4.93) | 36 (4.42) | 26 (3.93) | 23 (3.16) | 24 (3.62) | 34 (4.43) | 24 (3.50) | 14 (2.05) | 23 (3.59) | 16 (3.38) | 11 (3.33) | 11 (3.65) | 18 (4.97) | 20.89 | -1.03 (-2.42, 0.35) | 0.143 |
| Moderate preterm birth | 722 (5.41) | 20 (5.14) | 11 (3.25) | 23 (5.67) | 20 (5.63) | 30 (7.13) | 23 (5.05) | 34 (6.75) | 47 (7.63) | 22 (4.48) | 35 (5.07) | 49 (6.75) | 54 (6.49) | 58 (7.12) | 47 (7.10) | 27 (3.71) | 30 (4.52) | 35 (4.56) | 32 (4.66) | 30 (4.39) | 26 (4.06) | 25 (5.27) | 17 (5.15) | 16 (5.32) | 11 (3.04) | -40.90 | -1.08 (-2.74, 0.62) | 0.204 |
| Late preterm birth | 2003 (15.02) | 58 (14.91) | 50 (14.79) | 66 (16.26) | 58 (16.34) | 65 (15.44) | 61 (13.41) | 91 (18.06) | 79 (12.82) | 74 (15.07) | 130 (18.81) | 112 (15.43) | 136 (16.35) | 131 (16.07) | 96 (14.50) | 114 (15.66) | 109 (16.44) | 110 (14.32) | 94 (13.70) | 88 (12.87) | 82 (12.81) | 65 (13.71) | 41 (12.42) | 37 (12.29) | 56 (15.47) | 3.75 | -0.73 (-1.59, 0.09) | 0.078 |
| **sPTB-noPPROM** | | | | | | | | | | | | | | | | | | | | | | | | | | | | |
| All preterm birth | 1393 (10.44) | 74 (19.02) | 45 (13.31) | 54 (13.30) | 51 (14.37) | 66 (15.68) | 67 (14.73) | 70 (13.89) | 65 (10.55) | 49 (9.98) | 79 (11.43) | 83 (11.43) | 99 (11.90) | 78 (9.57) | 64 (9.67) | 54 (7.42) | 64 (9.65) | 69 (8.98) | 58 (8.45) | 46 (6.73) | 35 (5.47) | 40 (8.44) | 23 (6.97) | 25 (8.31) | 35 (9.67) | -49.18 | -2.62 (-4.09, -1.58) | < 0.001 |
| Extreme preterm birth | 132 (0.99) | 7 (1.80) | 5 (1.48) | 3 (0.74) | 4 (1.13) | 10 (2.38) | 7 (1.54) | 0 (0.00) | 4 (0.65) | 6 (1.22) | 6 (0.87) | 6 (0.83) | 8 (0.96) | 2 (0.25) | 6 (0.91) | 6 (0.82) | 8 (1.21) | 11 (1.43) | 9 (1.31) | 11 (1.61) | 3 (0.47) | 2 (0.42) | 5 (1.52) | 3 (1.00) | 0 (0.00) | -100.00 | -2.60 (-7.72, 2.80) | 0.323 |
| Very preterm birth | 236 (1.77) | 8 (2.06) | 9 (2.66) | 8 (1.97) | 11 (3.10) | 8 (1.90) | 12 (2.64) | 11 (2.18) | 16 (2.60) | 6 (1.22) | 14 (2.03) | 11 (1.52) | 17 (2.04) | 18 (2.21) | 10 (1.51) | 9 (1.24) | 13 (1.96) | 10 (1.30) | 9 (1.31) | 4 (0.58) | 6 (0.94) | 9 (1.90) | 4 (1.21) | 5 (1.66) | 8 (2.21) | 7.46 | -1.18 (-4.76, 1.33) | 0.359 |
| Moderate preterm birth | 259 (1.94) | 13 (3.34) | 4 (1.18) | 10 (2.46) | 9 (2.54) | 13 (3.09) | 9 (1.98) | 16 (3.17) | 14 (2.27) | 7 (1.43) | 8 (1.16) | 22 (3.03) | 22 (2.64) | 19 (2.33) | 15 (2.27) | 9 (1.24) | 11 (1.66) | 15 (1.95) | 11 (1.60) | 6 (0.88) | 5 (0.78) | 5 (1.05) | 6 (1.82) | 6 (1.99) | 4 (1.10) | -66.94 | -3.20 (-5.68, -0.69) | 0.014 |
| Late preterm birth | 766 (5.74) | 46 (11.83) | 27 (7.99) | 33 (8.13) | 27 (7.61) | 35 (8.31) | 39 (8.57) | 43 (8.53) | 31 (5.03) | 30 (6.11) | 51 (7.38) | 44 (6.06) | 52 (6.25) | 39 (4.79) | 33 (4.98) | 30 (4.12) | 32 (4.83) | 33 (4.30) | 29 (4.23) | 25 (3.65) | 21 (3.28) | 24 (5.06) | 8 (2.42) | 11 (3.65) | 23 (6.35) | -46.27 | -2.29 (-4.78, -0.93) | 0.001 |
| **PTB-PPROM** | | | | | | | | | | | | | | | | | | | | | | | | | | | | |
| All preterm birth | 2096 (15.72) | 28 (7.20) | 40 (11.83) | 53 (13.05) | 52 (14.65) | 58 (13.78) | 49 (10.77) | 76 (15.08) | 101 (16.40) | 79 (16.09) | 126 (18.23) | 124 (17.08) | 148 (17.79) | 153 (18.77) | 114 (17.22) | 121 (16.62) | 110 (16.59) | 123 (16.02) | 108 (15.74) | 100 (14.62) | 104 (16.25) | 74 (15.61) | 51 (15.45) | 47 (15.61) | 57 (15.75) | 118.75 | 3.05 (2.46, 3.72) | < 0.001 |
| Extreme preterm birth | 93 (0.70) | 1 (0.26) | 3 (0.89) | 3 (0.74) | 3 (0.85) | 3 (0.71) | 1 (0.22) | 4 (0.79) | 4 (0.65) | 3 (0.61) | 4 (0.58) | 6 (0.83) | 8 (0.96) | 4 (0.49) | 3 (0.45) | 5 (0.69) | 3 (0.45) | 2 (0.26) | 7 (1.02) | 3 (0.44) | 5 (0.78) | 6 (1.27) | 0 (0.00) | 5 (1.66) | 7 (1.93) | 652.21 | -13.00 (-40.69, 28.56) | 0.442 |
| Very preterm birth | 303 (2.27) | 8 (2.06) | 7 (2.07) | 4 (0.99) | 7 (1.97) | 8 (1.90) | 12 (2.64) | 6 (1.19) | 16 (2.60) | 17 (3.46) | 16 (2.32) | 23 (3.17) | 24 (2.88) | 18 (2.21) | 16 (2.42) | 14 (1.92) | 11 (1.66) | 24 (3.13) | 15 (2.19) | 10 (1.46) | 17 (2.66) | 7 (1.48) | 7 (2.12) | 6 (1.99) | 10 (2.76) | 34.32 | 0.62 (-1.28, 2.60) | 0.549 |
| Moderate preterm birth | 463 (3.47) | 7 (1.80) | 7 (2.07) | 13 (3.20) | 11 (3.10) | 17 (4.04) | 14 (3.08) | 18 (3.57) | 33 (5.36) | 15 (3.05) | 27 (3.91) | 27 (3.72) | 32 (3.85) | 39 (4.79) | 32 (4.83) | 18 (2.47) | 19 (2.87) | 20 (2.60) | 21 (3.06) | 24 (3.51) | 21 (3.28) | 20 (4.22) | 11 (3.33) | 10 (3.32) | 7 (1.93) | 7.46 | 2.10 (0.12, 4.16) | 0.040 |
| Late preterm birth | 1237 (9.27) | 12 (3.08) | 23 (6.80) | 33 (8.13) | 31 (8.73) | 30 (7.13) | 22 (4.84) | 48 (9.52) | 48 (7.79) | 44 (8.96) | 79 (11.43) | 68 (9.37) | 84 (10.10) | 92 (11.29) | 63 (9.52) | 84 (11.54) | 77 (11.61) | 77 (10.03) | 65 (9.48) | 63 (9.21) | 61 (9.53) | 41 (8.65) | 33 (10.00) | 26 (8.64) | 33 (9.12) | 195.51 | 4.34 (2.97, 5.81) | < 0.001 |
| **iPTB** | | | | | | | | | | | | | | | | | | | | | | | | | | | | |
| All preterm birth | 3250 (24.37) | 76 (19.54) | 69 (20.41) | 78 (19.21) | 67 (18.87) | 93 (22.09) | 109 (23.96) | 88 (17.46) | 152 (24.68) | 102 (20.77) | 133 (19.25) | 142 (19.56) | 135 (16.23) | 188 (23.07) | 155 (23.41) | 192 (26.37) | 161 (24.28) | 188 (24.48) | 205 (29.88) | 211 (30.85) | 203 (31.72) | 158 (33.33) | 100 (30.30) | 111 (36.88) | 134 (37.02) | 89.47 | 2.75 (1.82, 3.69) | < 0.001 |
| Extreme preterm birth | 54 (0.40) | 1 (0.26) | 1 (0.30) | 1 (0.25) | 1 (0.28) | 1 (0.24) | 4 (0.88) | 1 (0.20) | 2 (0.32) | 4 (0.81) | 1 (0.14) | 3 (0.41) | 2 (0.24) | 3 (0.37) | 2 (0.30) | 3 (0.41) | 2 (0.30) | 2 (0.26) | 5 (0.73) | 2 (0.29) | 4 (0.63) | 3 (0.63) | 0 (0.00) | 3 (1.00) | 3 (0.83) | 222.38 | -11.87 (-39.45, 29.66) | 0.470 |
| Very preterm birth | 303 (2.27) | 10 (2.57) | 6 (1.78) | 9 (2.22) | 7 (1.97) | 15 (3.56) | 12 (2.64) | 10 (1.98) | 19 (3.08) | 13 (2.65) | 12 (1.74) | 19 (2.62) | 11 (1.32) | 17 (2.09) | 13 (1.96) | 13 (1.79) | 14 (2.11) | 13 (1.69) | 14 (2.04) | 21 (3.07) | 16 (2.50) | 15 (3.16) | 7 (2.12) | 10 (3.32) | 7 (1.93) | -24.78 | 0.15 (-1.77, 2.09) | 0.898 |
| Moderate preterm birth | 493 (3.70) | 11 (2.83) | 13 (3.85) | 11 (2.71) | 13 (3.66) | 22 (5.23) | 15 (3.30) | 12 (2.38) | 31 (5.03) | 17 (3.46) | 29 (4.20) | 22 (3.03) | 32 (3.85) | 23 (2.82) | 24 (3.63) | 33 (4.53) | 14 (2.11) | 28 (3.65) | 27 (3.94) | 33 (4.82) | 24 (3.75) | 21 (4.43) | 15 (4.55) | 13 (4.32) | 10 (2.76) | -2.31 | 0.64 (-0.96, 2.28) | 0.437 |
| Late preterm birth | 2400 (18.00) | 54 (13.88) | 49 (14.50) | 57 (14.04) | 46 (12.96) | 55 (13.06) | 78 (17.14) | 65 (12.90) | 100 (16.23) | 68 (13.85) | 91 (13.17) | 98 (13.50) | 90 (10.82) | 145 (17.79) | 116 (17.52) | 143 (19.64) | 131 (19.76) | 145 (18.88) | 159 (23.18) | 155 (22.66) | 159 (24.84) | 119 (25.11) | 78 (23.64) | 85 (28.24) | 114 (31.49) | 126.86 | 3.39 (2.38, 4.45) | < 0.001 |

| **Supplementary table 6. Indication of all iPTB among singleton and twin pregnancies.** | | | | | | | | | | | | | | | | | | | | | | | | | | | | |
| --- | --- | --- | --- | --- | --- | --- | --- | --- | --- | --- | --- | --- | --- | --- | --- | --- | --- | --- | --- | --- | --- | --- | --- | --- | --- | --- | --- | --- |
|  | **Total** | **2000** | **2001** | **2002** | **2003** | **2004** | **2005** | **2006** | **2007** | **2008** | **2009** | **2010** | **2011** | **2012** | **2013** | **2014** | **2015** | **2016** | **2017** | **2018** | **2019** | **2020** | **2021** | **2022** | **2023** | **% Change 2000-2023** | **Average annual percent change (95% CI)** | **p-value** |
| **Singleton Pregnancy** | | | | | | | | | | | | | | | | | | | | | | | | | | | | |
|  | **n = 14190** | **n = 547** | **n = 559** | **n = 574** | **n = 568** | **n = 590** | **n = 640** | **n = 568** | **n = 605** | **n = 465** | **n = 652** | **n = 614** | **n = 644** | **n = 699** | **n = 621** | **n = 689** | **n = 669** | **n = 632** | **n = 625** | **n = 646** | **n = 603** | **n = 572** | **n = 510** | **n = 454** | **n = 444** |  | | |
| Hypertensive Disorders | 6311 (44.47) | 176 (32.18) | 197 (35.24) | 204 (35.54) | 199 (35.04) | 207 (35.08) | 241 (37.66) | 224 (39.44) | 239 (39.50) | 183 (39.35) | 266 (40.80) | 268 (43.65) | 299 (46.43) | 326 (46.64) | 299 (48.15) | 340 (49.35) | 317 (47.38) | 321 (50.79) | 293 (46.88) | 363 (56.19) | 321 (53.23) | 303 (52.97) | 273 (53.53) | 226 (49.78) | 226 (50.90) | 58.20 | 1.91 (1.41, 2.28) | < 0.001 |
| Antepartum Haemorrhage | 3706 (26.12) | 195 (35.65) | 170 (30.41) | 171 (29.79) | 171 (30.11) | 153 (25.93) | 153 (23.91) | 160 (28.17) | 154 (25.45) | 116 (24.95) | 173 (26.53) | 152 (24.76) | 169 (26.24) | 183 (26.18) | 159 (25.60) | 179 (25.98) | 155 (23.17) | 149 (23.58) | 179 (28.64) | 135 (20.90) | 147 (24.38) | 146 (25.52) | 124 (24.31) | 109 (24.01) | 104 (23.42) | -34.29 | -1.59 (-2.13, -0.88) | < 0.001 |
| Chorioamnionitis | 48 (0.34) | 0 (0.00) | 0 (0.00) | 0 (0.00) | 1 (0.18) | 0 (0.00) | 0 (0.00) | 0 (0.00) | 1 (0.17) | 1 (0.22) | 1 (0.15) | 0 (0.00) | 2 (0.31) | 0 (0.00) | 1 (0.16) | 1 (0.15) | 8 (1.20) | 3 (0.47) | 3 (0.48) | 8 (1.24) | 1 (0.17) | 5 (0.87) | 3 (0.59) | 5 (1.10) | 4 (0.90) | NA | 155.41 (52.36, 324.93) | < 0.001 |
| Abnormal Cardiotocogram | 1397 (9.84) | 56 (10.24) | 48 (8.59) | 39 (6.79) | 49 (8.63) | 64 (10.85) | 55 (8.59) | 51 (8.98) | 55 (9.09) | 38 (8.17) | 68 (10.43) | 66 (10.75) | 58 (9.01) | 77 (11.02) | 62 (9.98) | 62 (9.00) | 60 (8.97) | 76 (12.03) | 87 (13.92) | 66 (10.22) | 71 (11.77) | 58 (10.14) | 42 (8.24) | 43 (9.47) | 46 (10.36) | 1.20 | 0.85 (-0.15, 1.83 | 0.087 |
| Intrauterine Growth Restriction | 820 (5.78) | 39 (7.13) | 34 (6.08) | 32 (5.57) | 40 (7.04) | 29 (4.92) | 47 (7.34) | 32 (5.63) | 32 (5.29) | 20 (4.30) | 31 (4.75) | 29 (4.72) | 24 (3.73) | 26 (3.72) | 29 (4.67) | 22 (3.19) | 41 (6.13) | 47 (7.44) | 27 (4.32) | 36 (5.57) | 32 (5.31) | 35 (6.12) | 47 (9.22) | 44 (9.69) | 45 (10.14) | 42.15 | 1.14 (-0.64, 2.89) | 0.179 |
| Oligohydramnios | 120 (0.85) | 6 (1.10) | 7 (1.25) | 13 (2.26) | 8 (1.41) | 8 (1.36) | 6 (0.94) | 3 (0.53) | 2 (0.33) | 2 (0.43) | 6 (0.92) | 3 (0.49) | 2 (0.31) | 6 (0.86) | 3 (0.48) | 2 (0.29) | 5 (0.75) | 3 (0.47) | 2 (0.32) | 8 (1.24) | 7 (1.16) | 3 (0.52) | 6 (1.18) | 7 (1.54) | 2 (0.45) | -58.93 | -2.21 (-7.79, 4.06) | 0.386 |
| Red Cell Isoimmunisation | 4 (0.03) | 0 (0.00) | 0 (0.00) | 0 (0.00) | 0 (0.00) | 0 (0.00) | 0 (0.00) | 0 (0.00) | 0 (0.00) | 1 (0.22) | 0 (0.00) | 1 (0.16) | 1 (0.16) | 0 (0.00) | 0 (0.00) | 0 (0.00) | 0 (0.00) | 0 (0.00) | 0 (0.00) | 0 (0.00) | 0 (0.00) | 0 (0.00) | 0 (0.00) | 1 (0.22) | 0 (0.00) | NA | 8.78 (-28.75, 67.48) | 0.700 |
| Cholestasis | 23 (0.16) | 0 (0.00) | 0 (0.00) | 1 (0.17) | 0 (0.00) | 1 (0.17) | 2 (0.31) | 0 (0.00) | 0 (0.00) | 2 (0.43) | 1 (0.15) | 1 (0.16) | 0 (0.00) | 1 (0.14) | 2 (0.32) | 0 (0.00) | 2 (0.30) | 1 (0.16) | 1 (0.16) | 0 (0.00) | 3 (0.50) | 1 (0.17) | 1 (0.20) | 2 (0.44) | 1 (0.23) | NA | 72.11 (-7.00, 210.69) | 0.082 |
| Maternal Disease | 1761 (12.41) | 75 (13.71) | 103 (18.43) | 114 (19.86) | 100 (17.61) | 128 (21.69) | 136 (21.25) | 98 (17.25) | 122 (20.17) | 102 (21.94) | 106 (16.26) | 94 (15.31) | 89 (13.82) | 80 (11.44) | 66 (10.63) | 83 (12.05) | 81 (12.11) | 32 (5.06) | 33 (5.28) | 30 (4.64) | 21 (3.48) | 21 (3.67) | 14 (2.75) | 17 (3.74) | 16 (3.60) | -73.72 | -7.73 (-9.22, -5.98) | < 0.001 |
| **Twin Pregnancy** | | | | | | | | | | | | | | | | | | | | | | | | | | | | |
|  | **n = 3250** | **n = 76** | **n = 69** | **n = 78** | **n = 67** | **n = 93** | **n = 109** | **n = 88** | **n = 152** | **n = 102** | **n = 133** | **n = 142** | **n = 135** | **n = 188** | **n = 155** | **n = 192** | **n = 161** | **n = 188** | **n = 205** | **n = 211** | **n = 203** | **n = 158** | **n = 100** | **n = 111** | **n = 134** |  | | |
| Hypertensive Disorders | 1039 (31.97) | 15 (19.74) | 18 (26.09) | 12 (15.38) | 21 (31.34) | 18 (19.35) | 23 (21.10) | 22 (25.00) | 33 (21.71) | 28 (27.45) | 34 (25.56) | 40 (28.17) | 55 (40.74) | 67 (35.64) | 62 (40.00) | 75 (39.06) | 50 (31.06) | 69 (36.70) | 71 (34.63) | 83 (39.34) | 75 (36.95) | 60 (37.97) | 38 (38.00) | 33 (29.73) | 37 (27.61) | 39.90 | 1.74 (-0.30, 4.06) | 0.084 |
| Antepartum Haemorrhage | 316 (9.72) | 7 (9.21) | 7 (10.14) | 8 (10.26) | 8 (11.94) | 13 (13.98) | 10 (9.17) | 7 (7.95) | 19 (12.50) | 11 (10.78) | 12 (9.02) | 21 (14.79) | 12 (8.89) | 26 (13.83) | 16 (10.32) | 22 (11.46) | 11 (6.83) | 16 (8.51) | 22 (10.73) | 20 (9.48) | 16 (7.88) | 13 (8.23) | 4 (4.00) | 7 (6.31) | 8 (5.97) | -35.18 | -2.44 (-4.93, 0.20) | 0.061 |
| Chorioamnionitis | 6 (0.18) | 0 (0.00) | 0 (0.00) | 0 (0.00) | 0 (0.00) | 1 (1.08) | 0 (0.00) | 1 (1.14) | 0 (0.00) | 0 (0.00) | 0 (0.00) | 0 (0.00) | 0 (0.00) | 2 (1.06) | 0 (0.00) | 0 (0.00) | 0 (0.00) | 0 (0.00) | 0 (0.00) | 0 (0.00) | 0 (0.00) | 0 (0.00) | 0 (0.00) | 1 (0.90) | 1 (0.75) | NA | 133.33 (11.81, 377.91) | 0.043 |
| Abnormal Cardiotocogram | 230 (7.08) | 12 (15.79) | 8 (11.59) | 5 (6.41) | 6 (8.96) | 8 (8.60) | 10 (9.17) | 6 (6.82) | 11 (7.24) | 7 (6.86) | 11 (8.27) | 6 (4.23) | 12 (8.89) | 12 (6.38) | 8 (5.16) | 14 (7.29) | 10 (6.21) | 13 (6.91) | 15 (7.32) | 16 (7.58) | 13 (6.40) | 10 (6.33) | 3 (3.00) | 7 (6.31) | 7 (5.22) | -66.92 | -2.84 (-4.37, -1.28) | 0.001 |
| Twin Complications | 64 (1.97) | 0 (0.00) | 0 (0.00) | 4 (5.13) | 0 (0.00) | 2 (2.15) | 2 (1.83) | 3 (3.41) | 6 (3.95) | 5 (4.90) | 3 (2.26) | 6 (4.23) | 7 (5.19) | 2 (1.06) | 3 (1.94) | 2 (1.04) | 1 (0.62) | 2 (1.06) | 1 (0.49) | 4 (1.90) | 4 (1.97) | 2 (1.27) | 1 (1.00) | 3 (2.70) | 1 (0.75) | NA | 144.58 (53.52, 323.16) | 0.009 |
| Intrauterine Growth Restriction | 577 (17.75) | 7 (9.21) | 8 (11.59) | 15 (19.23) | 14 (20.90) | 14 (15.05) | 17 (15.60) | 10 (11.36) | 20 (13.16) | 14 (13.73) | 25 (18.80) | 19 (13.38) | 19 (14.07) | 28 (14.89) | 30 (19.35) | 33 (17.19) | 40 (24.84) | 35 (18.62) | 39 (19.02) | 41 (19.43) | 16 (17.73) | 25 (15.82) | 24 (24.00) | 30 (27.03) | 34 (25.37) | 175.48 | 2.62 (1.12, 4.13) | 0.001 |
| Oligohydramnios | 18 (0.55) | 0 (0.00) | 0 (0.00) | 2 (2.56) | 0 (0.00) | 0 (0.00) | 3 (2.75) | 0 (0.00) | 0 (0.00) | 0 (0.00) | 0 (0.00) | 1 (0.70) | 0 (0.00) | 3 (1.60) | 0 (0.00) | 2 (1.04) | 0 (0.00) | 1 (0.53) | 1 (0.49) | 0 (0.00) | 2 (0.99) | 3 (1.90) | 0 (0.00) | 0 (0.00) | 0 (0.00) | NA | 21.31 (-38.21, 136.44) | 0.607 |
| Red Cell Isoimmunisation | 0 (0.00) | 0 (0.00) | 0 (0.00) | 0 (0.00) | 0 (0.00) | 0 (0.00) | 0 (0.00) | 0 (0.00) | 0 (0.00) | 0 (0.00) | 0 (0.00) | 0 (0.00) | 0 (0.00) | 0 (0.00) | 0 (0.00) | 0 (0.00) | 0 (0.00) | 0 (0.00) | 0 (0.00) | 0 (0.00) | 0 (0.00) | 0 (0.00) | 0 (0.00) | 0 (0.00) | 0 (0.00) | NA | NA | NA |
| Cholestasis | 9 (0.28) | 0 (0.00) | 0 (0.00) | 0 (0.00) | 0 (0.00) | 0 (0.00) | 0 (0.00) | 1 (1.14) | 0 (0.00) | 0 (0.00) | 0 (0.00) | 1 (0.70) | 2 (1.48) | 1 (0.53) | 0 (0.00) | 1 (0.52) | 3 (1.86) | 0 (0.00) | 0 (0.00) | 0 (0.00) | 0 (0.00) | 0 (0.00) | 0 (0.00) | 0 (0.00) | 0 (0.00) | NA | -4.72 (-64.73, 148.96) | 0.877 |
| Maternal Disease | 225 (6.92) | 5 (6.58) | 7 (10.14) | 8 (10.26) | 5 (7.46) | 14 (15.05) | 12 (11.01) | 9 (10.23) | 18 (11.84) | 6 (5.88) | 15 (11.28) | 9 (6.34) | 6 (4.44) | 11 (5.85) | 11 (7.10) | 10 (5.21) | 12 (7.45) | 10 (5.32) | 12 (5.85) | 8 (3.79) | 9 (4.43) | 9 (5.70) | 4 (4.00) | 6 (5.41) | 9 (6.72) | 2.09 | -3.52 (-5.58, -1.49) | 0.001 |
| Twin Pregnancy | 766 (23.57) | 30 (39.47) | 21 (30.43) | 24 (30.77) | 13 (19.40) | 23 (24.73) | 32 (29.36) | 29 (32.95) | 45 (29.61) | 31 (30.39) | 33 (24.81) | 39 (27.46) | 22 (16.30) | 36 (19.15) | 25 (16.13) | 33 (17.19) | 34 (21.12) | 42 (22.34) | 44 (21.46) | 39 (18.48) | 48 (23.65) | 36 (22.78) | 26 (26.00) | 24 (21.62) | 37 (27.61) | -30.05 | -1.80 (-2.77, -0.66) | 0.003 |

Supplementary figure 2. The proportion of different indications for iPTB in a) singletons, b) twins

a)

b)

| **Supplementary table 7. The number and rate (per 1000 total births) of stillbirth and perinatal death among singleton and twin deliveries at < 37 weeks of gestation.** | | | | | | | | | | | | | | | | | | | | | | | | | | | | |
| --- | --- | --- | --- | --- | --- | --- | --- | --- | --- | --- | --- | --- | --- | --- | --- | --- | --- | --- | --- | --- | --- | --- | --- | --- | --- | --- | --- | --- |
|  | **Total** | **2000** | **2001** | **2002** | **2003** | **2004** | **2005** | **2006** | **2007** | **2008** | **2009** | **2010** | **2011** | **2012** | **2013** | **2014** | **2015** | **2016** | **2017** | **2018** | **2019** | **2020** | **2021** | **2022** | **2023** | **% Change 2000-2023** | **Average annual percent change (95% CI)** | **p-value** |
| **Singleton pregnancy** | | | | | | | | | | | | | | | | | | | | | | | | | | | | |
|  | **n = 56171** | **n = 2486** | **n = 2218** | **n = 2317** | **n = 2269** | **n = 2427** | **n = 2379** | **n = 2377** | **n = 2405** | **n = 2091** | **n = 2533** | **n = 2682** | **n = 2856** | **n = 2974** | **n = 2430** | **n = 2701** | **n = 2458** | **n = 2549** | **n = 2549** | **n = 2405** | **n = 2289** | **n = 1915** | **n = 1735** | **n = 1579** | **n = 1547** |  | | |
| **Singleton - Stillbirth** | | | | | | | | | | | | | | | | | | | | | | | | | | | | |
| All preterm birth | 1782 (31.72) | 86 (34.59) | 71 (32.01) | 86 (37.12) | 82 (36.14) | 61 (25.13) | 88 (36.99) | 60 (25.24) | 70 (29.11) | 61 (29.17) | 81 (31.98) | 70 (26.10) | 93 (32.56) | 86 (28.92) | 78 (32.10) | 72 (26.66) | 82 (33.36) | 84 (32.95) | 92 (36.09) | 67 (27.86) | 68 (29.71) | 72 (37.60) | 72 (41.50) | 47 (29.77) | 53 (34.26) | -0.97 | 0.24 (-0.84, 1.29) | 0.656 |
| Extreme preterm birth | 578 (197.00) | 38 (294.57) | 24 (195.12) | 28 (259.26) | 28 (252.25) | 23 (188.52) | 23 (181.10) | 18 (174.76) | 26 (196.97) | 19 (204.30) | 29 (250.00) | 23 (175.57) | 29 (192.05) | 24 (152.87) | 23 (184.00) | 21 (156.72) | 26 (174.50) | 23 (166.67) | 35 (257.35) | 15 (133.93) | 18 (134.33) | 23 (212.96) | 34 (311.93) | 13 (128.71) | 15 (176.47) | -40.09 | -1.28 (02.85, 0.28) | 0.105 |
| Very preterm birth | 519 (86.51) | 27 (108.00) | 19 (85.20) | 29 (116.00) | 20 (90.91) | 16 (61.78) | 25 (103.31) | 16 (80.00) | 16 (59.26) | 15 (66.37) | 24 (87.91) | 16 (61.07) | 29 (97.32) | 24 (82.19) | 25 (83.61) | 22 (72.61) | 26 (88.14) | 20 (71.17) | 28 (98.59) | 23 (84.25) | 24 (94.86) | 21 (99.06) | 23 (113.86) | 15 (88.24) | 16 (98.77) | -8.55 | -0.19 (-1.70, 1.70) | 0.803 |
| Moderate preterm birth | 269 (37.35) | 7 (23.18) | 13 (48.51) | 5 (19.08) | 12 (38.96) | 7 (25.09) | 19 (69.34) | 9 (30.72) | 4 (13.25) | 10 (37.31) | 10 (33.78) | 15 (41.90) | 17 (48.02) | 14 (36.94) | 11 (35.03) | 14 (34.991) | 9 (27.69) | 21 (59.32) | 12 (34.99) | 10 (30.21) | 9 (31.58) | 13 (50.39) | 7 (29.41) | 11 (53.66) | 10 (48.54) | 109.43 | 1.60 (-0.94, 4.16) | 0.207 |
| Late preterm birth | 416 (10.39) | 14 (7.76) | 15 (9.35) | 24 (14.14) | 22 (13.50) | 15 (8.49) | 21 (12.10) | 17 (9.55) | 24 (14.11) | 17 (11.30) | 18 (9.74) | 16 (8.29) | 18 (8.77) | 24 (11.18) | 19 (11.23) | 15 (8.05) | 21 (12.43) | 20 (11.26) | 17 (9.52) | 19 (11.25) | 17 (10.51) | 15 (11.22) | 8 (6.75) | 8 (7.25) | 12 (10.97) | 41.42 | -0.59 (-2.05, 0.83) | 0.396 |
| **Singleton - Perinatal Death** | | | | | | | | | | | | | | | | | | | | | | | | | | | | |
| All preterm birth | 2305 (41.04) | 113 (45.45) | 92 (41.48) | 106 (45.75) | 98 (43.19) | 85 (35.02) | 117 (49.18) | 78 (32.81) | 90 (37.42) | 84 (40.17) | 105 (41.45) | 101 (37.66) | 115 (40.27) | 115 (38.67) | 109 (44.86) | 95 (35.17) | 109 (44.34) | 110 (43.15) | 112 (43.94) | 84 (34.93) | 86 (37.57) | 91 (47.52) | 84 (48.41) | 64 (40.53) | 62 (40.08) | -11.83 | 0.03 (-0.85, 0.92) | 0.957 |
| Extreme preterm birth | 824 (280.85) | 48 (372.09) | 34 (276.42) | 32 (296.30) | 32 (288.29) | 34 (278.69) | 33 (259.84) | 29 (281.55) | 33 (250.00) | 29 (311.83) | 36 (310.34) | 39 (297.71) | 28 (251.66) | 42 (267.52) | 38 (304.00) | 34 (253.73) | 41 (275.17) | 36 (260.87) | 47 (345.59) | 22 (196.43) | 30 (223.88) | 30 (277.78) | 42 (385.32) | 24 (237.62) | 21 (247.06) | -33.60 | -0.62 (-1.60, 0.38) | 0.218 |
| Very preterm birth | 649 (108.18) | 36 (144.00) | 26 (116.59) | 42 (168.00) | 28 (127.27) | 24 (92.66) | 30 (123.97) | 17 (85.00) | 22 (81.48) | 19 (84.07) | 33 (120.88) | 22 (83.97) | 33 (110.74) | 26 (89.04) | 34 (113.71) | 27 (89.11) | 33 (111.86) | 23 (81.85) | 32 (112.68) | 30 (109.89) | 28 (110.67) | 25 (117.92) | 25 (123.76) | 17 (100.00) | 17 (104.94) | -27.13 | -1.14 (-2.60, 0.72) | 0.187 |
| Moderate preterm birth | 321 (44.56) | 11 (36.42) | 15 (55.97) | 6 (22.90) | 13 (42.21) | 8 (28.67) | 25 (91.24) | 12 (40.96) | 5 (16.56) | 13 (48.51) | 13 (43.92) | 18 (50.28) | 20 (56.50) | 21 (55.41) | 12 (38.22) | 16 (39.90) | 11 (33.85) | 23 (64.97) | 14 (40.82) | 11 (33.23) | 9 (31.58) | 15 (58.14) | 8 (33.61) | 12 (58.54) | 10 (48.54) | 33.27 | 0.74 (-1.71, 3.20) | 0.572 |
| Late preterm birth | 511 (12.76) | 18 (9.97) | 17 (10.60) | 26 (15.32) | 25 (15.34) | 19 (10.75) | 29 (16.71) | 20 (11.23) | 30 (17.64) | 23 (15.29) | 23 (12.45) | 22 (11.39) | 24 (11.69) | 26 (12.12) | 25 (14.78) | 18 (9.66) | 24 (14.21) | 28 (15.77) | 19 (10.64) | 21 (12.43) | 19 (11.75) | 21 (15.71) | 9 (7.59) | 11 (9.97) | 14 (12.80) | 28.33 | -0.59 (-2.31, 1.14) | 0.458 |
| **Twin pregnancy** | | | | | | | | | | | | | | | | | | | | | | | | | | | | |
|  | **n = 13544** | **n = 358** | **n = 310** | **n = 374** | **n = 342** | **n = 438** | **n = 454** | **n = 468** | **n = 642** | **n = 464** | **n = 680** | **n = 706** | **n = 766** | **n = 844** | **n = 666** | **n = 736** | **n = 672** | **n = 764** | **n = 748** | **n = 714** | **n = 686** | **n = 544** | **n = 348** | **n = 366** | **n = 454** |  | | |
| **Twin - Stillbirth** | | | | | | | | | | | | | | | | | | | | | | | | | | | | |
| All preterm birth | 180 (13.29) | 6 (16.76) | 5 (16.13) | 9 (24.06) | 3 (8.77) | 11 (25.11) | 10 (22.03) | 4 (8.55) | 15 (23.36) | 7 (15.09) | 16 (23.53) | 17 (24.08) | 5 (6.53) | 12 (14.22) | 5 (7.51) | 8 (10.87) | 7 (10.42) | 8 (10.47) | 7 (9.36) | 7 (9.80) | 5 (7.29) | 3 (5.51) | 2 (5.75) | 2 (5.46) | 6 (13.22) | -21.15 | -4.58 (-7.48, -1.62) | 0.003 |
| Extreme preterm birth | 44 (75.09) | 0 (0.00) | 3 (150.00) | 0 (0.00) | 3 (166.67) | 5 (166.67) | 3 (115.38) | 0 (0.00) | 3 (136.36) | 3 (107.14) | 5 (192.31) | 4 (117.65) | 0 (0.00) | 2 (100.00) | 0 (0.00) | 2 (66.67) | 1 (38.46) | 2 (66.67) | 2 (45.45) | 3 (93.75) | 2 (76.92) | 0 (0.00) | 1 (100.00) | 0 (0.00) | 0 (0.00) | NA | -3.99 (-8.20, 0.40) | 0.072 |
| Very preterm birth | 48 (28.17) | 2 (37.04) | 0 (0.00) | 3 (68.18) | 0 (0.00) | 1 (16.13) | 1 (13.89) | 0 (0.00) | 8 (75.47) | 1 (13.89) | 5 (59.52) | 7 (64.81) | 1 (9.62) | 3 (27.78) | 0 (0.00) | 3 (41.67) | 2 (25.64) | 2 (20.83) | 4 (50.00) | 1 (14.29) | 2 (25.64) | 1 (116.13) | 0 (0.00) | 0 (0.00) | 1 (20.00) | -46.00 | -1.05 (-6.15, 4.26) | 0.661 |
| Moderate preterm birth | 25 (10.25) | 0 (0.00) | 0 (0.00) | 2 (28.57) | 0 (0.00) | 4 (37.74) | 3 (38.46) | 2 (21.74) | 2 (12.82) | 0 (0.00) | 3 (23.44) | 0 (0.00) | 2 (11.63) | 0 (0.00) | 1 (7.04) | 0 (0.00) | 2 (22.73) | 3 (23.44) | 0 (0.00) | 0 (0.00) | 0 (0.00) | 1 (10.87) | 0 (0.00) | 0 (0.00) | 0 (0.00) | NA | -3.63 (-9.96, 2.81) | 0.232 |
| Late preterm birth | 63 (7.15) | 4 (17.86) | 2 (10.10) | 4 (16.26) | 0 (0.00) | 1 (4.17) | 3 (10.79) | 2 (6.41) | 2 (5.59) | 3 (10.49) | 3 (6.79) | 6 (14.22) | 2 (4.41) | 7 (12.64) | 4 (9.43) | 3 (5.84) | 2 (4.17) | 1 (1.96) | 1 (1.98) | 3 (6.17) | 1 (2.07) | 1 (2.72) | 1 (4.20) | 2 (8.20) | 5 (14.62) | -18.13 | 0.72 (-6.78, 5.49) | 0.855 |
| **Twin - Perinatal Death** | | | | | | | | | | | | | | | | | | | | | | | | | | | | |
| All preterm birth | 287 (21.19) | 15 (41.90) | 8 (25.81) | 11 (29.41) | 10 (29.24) | 16 (36.53) | 16 (35.24) | 8 (17.09) | 21 (32.71) | 11 (23.71) | 22 (32.35) | 20 (28.33) | 16 (20.89) | 14 (16.59) | 5 (7.51) | 11 (14.95) | 10 (14.88) | 13 (17.02) | 15 (20.05) | 10 (14.01) | 10 (14.58) | 5 (9.19) | 7 (20.11) | 5 (13.66) | 8 (17.62) | -57.94 | -4.31 (-6.55, -2.00) | < 0.001 |
| Extreme preterm birth | 113 (192.83) | 6 (333.33) | 4 (200.00) | 1 (71.43) | 6 (333.33) | 9 (300.00) | 7 (269.23) | 1 (100.00) | 8 (363.64) | 4 (142.86) | 10 (384.62) | 5 (147.06) | 8 (222.22) | 4 (200.00) | 0 (0.00) | 5 (166.67) | 3 (115.38) | 5 (166.67) | 8 (181.82) | 6 (187.50) | 4 (153.85) | 2 (90.91) | 2 (200.00) | 3 (136.36) | 2 (100.00) | -70.00 | -2.78 (-6.89, 1.48) | 0.196 |
| Very preterm birth | 65 (38.15) | 5 (92.59) | 1 (22.73) | 3 (68.18) | 1 (20.00) | 1 (16.13) | 1 (13.89) | 2 (37.04) | 8 (75.47) | 2 (27.78) | 6 (71.43) | 7 (64.81) | 3 (28.85) | 3 (27.78) | 0 (0.00) | 3 (41.67) | 2 (25.64) | 3 (31.25) | 5 (62.50) | 1 (14.29) | 3 (38.46) | 1 (16.13) | 3 (83.33) | 0 (0.00) | 1 (20.00) | -78.40 | -2.05 (-7.45, 3.54) | 0.419 |
| Moderate preterm birth | 38 (15.59) | 0 (0.00) | 1 (20.83) | 2 (28.57) | 3 (45.45) | 4 (37.74) | 4 (51.28) | 3 (32.61) | 3 (19.23) | 1 (12.82) | 3 (23.44) | 0 (0.00) | 3 (17.44) | 0 (0.00) | 1 (7.04) | 0 (0.00) | 2 (22.73) | 4 (31.25) | 0 (0.00) | 0 (0.00) | 2 (20.00) | 1 (10.87) | 1 (15.63) | 0 (0.00) | 0 (0.00) | NA | -4.69 (-9.54, 0.30) | 0.068 |
| Late preterm birth | 71 (8.05) | 4 (17.86) | 2 (10.10) | 5 (20.33) | 0 (0.00) | 2 (8.33) | 4 (14.39) | 2 (6.41) | 2 (5.59) | 4 (13.99) | 3 (6.79) | 8 (18.96) | 2 (4.41) | 7 (12.64) | 4 (9.43) | 3 (5.84) | 3 (6.25) | 1 (1.96) | 2 (3.95) | 3 (6.17) | 1 (2.07) | 1 (2.72) | 1 (4.20) | 2 (8.20) | 5 (14.62) | -18.13 | -3.87 (-8.62, 0.97) | 0.113 |

| **Supplementary table 8. The number and rate (per 1000 livebirth) of neonatal death among singleton deliveries at < 37 weeks of gestation.** | | | | | | | | | | | | | | | | | | | | | | | | | | | | |
| --- | --- | --- | --- | --- | --- | --- | --- | --- | --- | --- | --- | --- | --- | --- | --- | --- | --- | --- | --- | --- | --- | --- | --- | --- | --- | --- | --- | --- |
|  | **Total (n = 54389)** | **2000 (n = 2400)** | **2001 (n = 2147)** | **2002 (n = 2231)** | **2003 (n = 2187)** | **2004 (n = 2366)** | **2005 (n = 2291)** | **2006 (n = 2317)** | **2007 (n = 2335)** | **2008 (n = 2030)** | **2009 (n = 2452)** | **2010 (n = 2612)** | **2011 (n = 2763)** | **2012 (n = 2888)** | **2013 (n = 2352)** | **2014 (n = 2629)** | **2015 (n = 2376)** | **2016 (n = 2465)** | **2017 (n = 2457)** | **2018 (n = 2338)** | **2019 (n = 2221)** | **2020 (n = 1843)** | **2021 (n = 1663)** | **2022 (n = 1532)** | **2023 (n = 1494)** | **% Change 2000-2023** | **Average annual percent change (95% CI)** | **p-value** |
| **Overall** | | | | | | | | | | | | | | | | | | | | | | | | | | | | |
| All preterm birth | 667 (12.26) | 32 (13.33) | 28 (13.04) | 22 (9.86) | 22 (10.06) | 30 (12.68) | 36 (15.71) | 23 (9.93) | 27 (11.56) | 34 (16.75) | 27 (11.01) | 43 (16.46) | 28 (10.13) | 38 (13.16) | 40 (17.01) | 30 (11.41) | 32 (13.47) | 28 (11.36) | 32 (13.02) | 24 (10.27) | 23 (10.36) | 23 (12.48) | 15 (9.02) | 20 (13.05) | 10 (6.69) | -49.80 | -0.89 (-2.45, 0.65) | 0.247 |
| Extreme preterm birth | 319 (135.40) | 12 (131.87) | 13 (131.31) | 6 (75.00) | 7 (84.34) | 15 (151.52) | 14 (134.62) | 12 (141.18) | 11 (103.77) | 15 (202.70) | 8 (91.95) | 24 (222.22) | 11 (90.16) | 24 (180.45) | 20 (196.08) | 17 (150.44) | 17 (138.21) | 13 (113.04) | 20 (198.02) | 9 (92.78) | 15 (129.78) | 7 (82.35) | 10 (133.33) | 13 (147.73) | 6 (85.71) | -35.00 | 0.02 (-2.74, 2.73) | 0.999 |
| Very preterm birth | 160 (29.20) | 9 (40.36) | 9 (44.12) | 13 (58.82) | 10 (50.00) | 8 (32.92) | 6 (27.65) | 2 (10.87) | 8 (31.50) | 5 (23.70) | 10 (40.16) | 7 (28.46) | 6 (22.30) | 3 (11.19) | 12 (43.80) | 6 (21.35) | 8 (29.74) | 4 (15.33) | 6 (23.44) | 10 (40.00) | 5 (21.83) | 5 (26.18) | 3 (16.76) | 3 (19.35) | 2 (13.70) | -66.06 | -3.31 (-5.79, -0.76) | 0.013 |
| Moderate preterm birth | 64 (9.23) | 5 (16.95) | 2 (7.84) | 1 (3.89) | 1 (3.38) | 1 (3.68) | 7 (27.45) | 5 (17.61) | 2 (6.71) | 5 (19.38) | 3 (10.49) | 3 (8.75) | 4 (11.87) | 7 (19.18) | 1 (3.30) | 3 (7.75) | 3 (9.49) | 3 (9.01) | 2 (6.04) | 1 (3.12) | 0 (0.00) | 3 (12.24) | 1 (4.33) | 1 (5.15) | 0 (0.00) | -100.00 | -3.65 (-8.61, 1.39) | 0.148 |
| Late preterm birth | 124 (3.13) | 6 (3.35) | 4 (2.52) | 2 (1.20) | 4 (2.49) | 6 (3.42) | 9 (5.25) | 4 (2.27) | 6 (3.58) | 9 (6.05) | 6 (3.28) | 9 (4.70) | 7 (3.44) | 4 (1.89) | 7 (4.18) | 4 (2.16) | 4 (2.40) | 8 (4.56) | 4 (2.26) | 4 (2.40) | 3 (1.88) | 8 (6.05) | 1 (0.85) | 3 (2.74) | 2 (1.85) | -44.82 | -1.29 (-4.31, 1.77) | 0.394 |
| **sPTB-noPPROM** | | | | | | | | | | | | | | | | | | | | | | | | | | | | |
| All preterm birth | 261 (13.76) | 13 (10.42) | 16 (16.91) | 10 (11.11) | 9 (9.54) | 12 (11.99) | 16 (17.70) | 7 (7.22) | 11 (12.39) | 11 (14.10) | 13 (14.22) | 18 (20.02) | 12 (13.00) | 14 (14.72) | 15 (20.63) | 14 (17.48) | 9 (13.31) | 10 (14.22) | 13 (18.73) | 9 (14.02) | 10 (16.72) | 4 (7.97) | 2 (4.14) | 9 (20.04) | 4 (9.26) | -11.11 | -0.28 (-2.87, 2.37) | 0.805 |
| Extreme preterm birth | 166 (143.47) | 8 (153.85) | 9 (155.17) | 4 (93.02) | 4 (100.00) | 7 (148.94) | 10 (188.68) | 4 (102.56) | 5 (111.11) | 6 (171.43) | 7 (162.79) | 16 (253.97) | 6 (101.69) | 12 (187.50) | 9 (176.47) | 10 (166.67) | 6 (100.00) | 4 (74.07) | 11 (275.00) | 4 (81.63) | 9 (166.67) | 3 (75.00) | 2 (58.82) | 7 (170.73) | 3 (90.91) | -40.91 | -1.29 (-4.41, 1.78) | 0.376 |
| Very preterm birth | 41 (21.29) | 1 (8.70) | 4 (50.63) | 6 (71.43) | 2 (26.67) | 3 (33.71) | 2 (25.64) | 1 (14.29) | 1 (12.99) | 2 (23.81) | 3 (33.33) | 1 (13.51) | 2 (21.51) | 0 (0.00) | 3 (30.00) | 1 (10.10) | 1 (11.24) | 3 (33.71) | 0 (0.00) | 3 (39.47) | 1 (14.93) | 0 (0.00) | 0 (0.00) | 0 (0.00) | 1 (18.52) | 112.96 | -3.88 (-8.27, 0.64) | 0.093 |
| Moderate preterm birth | 21 (9.40) | 2 (15.04) | 1 (10.99) | 0 (0.00) | 1 (9.62) | 0 (0.00) | 2 (24.39) | 1 (9.71) | 1 (10.10) | 0 (0.00) | 1 (9.90) | 1 (9.01) | 2 (19.42) | 0 (0.00) | 0 (0.00) | 2 (17.39) | 1 (9.43) | 1 (10.10) | 2 (22.73) | 1 (10.64) | 0 (0.00) | 1 (14.71) | 0 (0.00) | 1 (16.39) | 0 (0.00) | -100.00 | 0.55 (-3.38, 4.52) | 0.786 |
| Late preterm birth | 33 (2.42) | 2 (2.11) | 2 (2.79) | 0 (0.00) | 2 (2.76) | 2 (2.61) | 2 (2.89) | 1 (1.32) | 4 (6.00) | 3 (5.18) | 2 (2.94) | 0 (0.00) | 2 (2.99) | 2 (2.99) | 3 (6.26) | 1 (1.90) | 1 (2.38) | 2 (4.34) | 0 (0.00) | 1 (2.36) | 0 (0.00) | 0 (0.00) | 0 (0.00) | 1 (3.36) | 0 (0.00) | -100.00 | -0.77 (-4.43, 3.03) | 0.672 |
| **PTB-PPROM** | | | | | | | | | | | | | | | | | | | | | | | | | | | | |
| All preterm birth | 171 (8.29) | 4 (8.33) | 3 (4.69) | 2 (2.65) | 3 (4.44) | 8 (10.32) | 10 (13.42) | 8 (10.26) | 6 (7.14) | 14 (17.86) | 5 (5.64) | 13 (11.84) | 7 (5.85) | 10 (8.08) | 14 (13.94) | 9 (7.91) | 13 (12.61) | 7 (6.49) | 8 (7.63) | 5 (5.05) | 5 (5.22) | 6 (8.40) | 3 (4.82) | 6 (10.29) | 2 (3.54) | -57.52 | -0.35 (-3.90, 3.29) | 0.829 |
| Extreme preterm birth | 90 (115.53) | 2 (105.26) | 2 (76.92) | 1 (45.45) | 2 (71.43) | 5 (135.14) | 3 (93.75) | 5 (185.19) | 3 (76.92) | 7 (225.81) | 0 (0.00) | 5 (151.52) | 4 (93.02) | 6 (133.33) | 6 (200.00) | 5 (147.06) | 9 (214.29) | 3 (69.77) | 5 (131.58) | 3 (90.91) | 3 (78.95) | 3 (90.91) | 2 (76.92) | 5 (142.86) | 1 (50.00) | -52.50 | 5.64 (-36.93, 77.47) | 0.744 |
| Very preterm birth | 36 (22.78) | 1 (28.57) | 1 (26.32) | 0 (0.00) | 1 (16.13) | 1 (14.08) | 2 (37.74) | 0 (0.00) | 2 (25.97) | 1 (13.70) | 5 (66.67) | 2 (23.53) | 1 (11.76) | 2 (29.41) | 5 (66.67) | 1 (10.87) | 3 (33.33) | 1 (12.66) | 3 (41.10) | 1 (14.08) | 2 (26.67) | 0 (0.00) | 0 (0.00) | 1 (20.83) | 0 (0.00) | -100.00 | -46.92 (-78.33, 54.01) | 0.139 |
| Moderate preterm birth | 14 (6.01) | 0 (0.00) | 0 (0.00) | 0 (0.00) | 0 (0.00) | 0 (0.00) | 2 (23.81) | 1 (12.20) | 0 (0.00) | 3 (30.93) | 0 (0.00) | 2 (14.71) | 1 (7.94) | 2 (14.81) | 1 (9.62) | 0 (0.00) | 0 (0.00) | 0 (0.00) | 0 (0.00) | 0 (0.00) | 0 (0.00) | 1 (13.33) | 1 (12.66) | 0 (0.00) | 0 (0.00) | NA | 4.56 (-46.52, 110.94) | 0.950 |
| Late preterm birth | 31 (1.95) | 1 (2.73) | 0 (0.00) | 1 (1.71) | 0 (0.00) | 2 (3.51) | 3 (5.21) | 2 (3.24) | 1 (1.61) | 3 (5.15) | 0 (0.00) | 4 (4.74) | 1 (1.06) | 0 (0.00) | 2 (2.52) | 3 (3.45) | 1 (1.25) | 3 (3.58) | 0 (0.00) | 1 (1.29) | 0 (0.00) | 2 (3.66) | 0 (0.00) | 0 (0.00) | 1 (2.28) | -16.44 | -20.86 (-58.39, 45.63) | 0.463 |
| **iPTB** | | | | | | | | | | | | | | | | | | | | | | | | | | | | |
| All preterm birth | 234 (15.84) | 15 (22.35) | 9 (16.10) | 10 (17.42) | 10 (17.61) | 10 (16.95) | 10 (15.63) | 8 (14.08) | 10 (16.53) | 9 (19.35) | 9 (13.80) | 12 (19.54) | 9 (13.98) | 13 (18.60) | 11 (17.71) | 7 (10.16) | 10 (14.95) | 11 (16.11) | 11 (15.38) | 10 (14.16) | 8 (12.03) | 13 (20.73) | 10 (17.95) | 5 (10.00) | 4 (8.08) | -63.85 | -3.46 (-5.27, -1.27) | 0.019 |
| Extreme preterm birth | 63 (150.00) | 2 (100.00) | 2 (133.33) | 1 (66.67) | 1 (66.67) | 3 (200.00) | 1 (52.63) | 3 (157.89) | 3 (136.36) | 2 (250.00) | 1 (52.63) | 3 (250.00) | 1 (50.00) | 6 (250.00) | 5 (238.10) | 2 (105.26) | 2 (95.24) | 6 (333.33) | 4 (173.91) | 2 (133.33) | 3 (125.00) | 1 (83.33) | 6 (400.00) | 1 (83.33) | 2 (117.65) | 17.65 | 1.93 (-3.29, 7.08) | 0.424 |
| Very preterm birth | 83 (42.07) | 7 (95.89) | 4 (45.98) | 7 (88.61) | 7 (111.11) | 4 (48.19) | 2 (23.53) | 1 (16.67) | 5 (50.00) | 2 (37.04) | 2 (23.81) | 4 (45.98) | 3 (32.97) | 1 (10.53) | 4 (40.40) | 4 (44.44) | 4 (44.44) | 0 (0.00) | 3 (29.41) | 6 (58.25) | 2 (22.99) | 5 (61.73) | 3 (40.54) | 2 (34.48) | 1 (18.18) | -81.04 | -11.50 (-45.26, 44.59) | 0.562 |
| Moderate preterm birth | 29 (12.25) | 3 (29.41) | 1 (11.24) | 1 (11.90) | 0 (0.00) | 1 (13.16) | 3 (33.71) | 3 (30.30) | 1 (10.53) | 2 (25.32) | 2 (20.41) | 0 (0.00) | 1 (9.26) | 5 (43.10) | 0 (0.00) | 1 (7.75) | 2 (18.35) | 2 (17.24) | 0 (0.00) | 0 (0.00) | 0 (0.00) | 1 (9.80) | 0 (0.00) | 0 (0.00) | 0 (0.00) | -100.00 | -58.47 (-80.13, -14.46) | 0.018 |
| Late preterm birth | 59 (5.89) | 3 (6.30) | 2 (5.43) | 1 (2.53) | 2 (5.12) | 2 (4.81) | 4 (8.95) | 1 (2.56) | 1 (2.58) | 3 (9.26) | 4 (8.87) | 5 (11.90) | 4 (9.41) | 1 (2.16) | 2 (5.01) | 0 (0.00) | 2 (4.45) | 3 (6.58) | 4 (8.60) | 2 (4.25) | 3 (6.74) | 6 (13.89) | 1 (2.64) | 2 (5.59) | 1 (2.82) | -55.18 | -4.53 (-39.36, 51.18) | 0.726 |

| **Supplementary table 9. The number and rate (per 1000 livebirth) of neonatal death among twin deliveries at < 37 weeks of gestation.** | | | | | | | | | | | | | | | | | | | | | | | | | | | | |
| --- | --- | --- | --- | --- | --- | --- | --- | --- | --- | --- | --- | --- | --- | --- | --- | --- | --- | --- | --- | --- | --- | --- | --- | --- | --- | --- | --- | --- |
|  | **Total (n = 13364)** | **2000 (n = 352)** | **2001 (n = 305)** | **2002 (n = 365)** | **2003 (n = 339)** | **2004 (n = 427)** | **2005 (n = 444)** | **2006 (n = 464)** | **2007 (n = 627)** | **2008 (n = 457)** | **2009 (n = 664)** | **2010 (n = 689)** | **2011 (n = 761)** | **2012 (n = 832)** | **2013 (n = 661)** | **2014 (n = 728)** | **2015 (n = 665)** | **2016 (n = 756)** | **2017 (n = 741)** | **2018 (n = 707)** | **2019 (n = 681)** | **2020 (n = 541)** | **2021 (n = 346)** | **2022 (n = 364)** | **2023 (n = 448)** | **% Change 2000-2023** | **Average annual percent change (95% CI)** | **p-value** |
| **Overall** | | | | | | | | | | | | | | | | | | | | | | | | | | | | |
| All preterm birth | 140 (10.48) | 9 (25.57) | 3 (9.84) | 2 (5.48) | 7 (20.65) | 7 (16.39) | 8 (18.02) | 5 (10.78) | 8 (12.76) | 6 (13.13) | 8 (12.05) | 6 (8.71) | 11 (14.45) | 3 (3.61) | 3 (4.54) | 3 (4.12) | 7 (10.53) | 7 (9.26) | 10 (13.50) | 8 (11.32) | 5 (7.34) | 4 (7.39) | 5 (14.45) | 3 (8.24) | 2 (4.46) | -82.54 | -3.07 (-5.97, -0.07) | 0.047 |
| Extreme preterm birth | 86 (158.67) | 6 (333.33) | 1 (58.82) | 1 (71.43) | 3 (200.00) | 6 (240.00) | 5 (217.39) | 1 (100.00) | 6 (315.79) | 2 (80.00) | 6 (285.71) | 2 (66.67) | 8 (222.22) | 3 (166.67) | 2 (90.91) | 3 (107.14) | 4 (160.00) | 4 (142.86) | 7 (166.67) | 5 (172.41) | 2 (83.33) | 3 (136.36) | 1 (111.11) | 3 (136.36) | 2 (100.00) | -70.00 | -1.21 (-4.65, 2.37) | 0.473 |
| Very preterm birth | 25 (15.10) | 3 (57.69) | 1 (22.73) | 0 (0.00) | 1 (20.00) | 0 (0.00) | 0 (0.00) | 2 (37.04) | 0 (0.00) | 2 (28.17) | 1 (12.66) | 1 (9.90) | 2 (19.42) | 0 (0.00) | 1 (12.82) | 0 (0.00) | 1 (13.16) | 2 (21.28) | 2 (26.32) | 1 (14.49) | 1 (13.16) | 1 (16.39) | 3 (83.33) | 0 (0.00) | 0 (0.00) | -100.00 | -0.15 (-5.22, 4.98) | 0.921 |
| Moderate preterm birth | 16 (6.63) | 0 (0.00) | 1 (20.83) | 0 (0.00) | 3 (45.45) | 0 (0.00) | 1 (13.33) | 1 (11.11) | 2 (12.99) | 1 (12.82) | 1 (8.00) | 0 (0.00) | 1 (5.88) | 0 (0.00) | 0 (0.00) | 0 (0.00) | 0 (0.00) | 1 (8.00) | 0 (0.00) | 1 (7.94) | 2 (20.00) | 0 (0.00) | 1 (15.63) | 0 (0.00) | 0 (0.00) | NA | -1.18 (-6.46, 3.99) | 0.641 |
| Late preterm birth | 13 (1.49) | 0 (0.00) | 0 (0.00) | 1 (4.13) | 0 (0.00) | 1 (4.18) | 2 (7.27) | 1 (3.23) | 0 (0.00) | 1 (3.53) | 0 (0.00) | 3 (7.21) | 0 (0.00) | 0 (0.00) | 0 (0.00) | 0 (0.00) | 2 (4.18) | 0 (0.00) | 1 (1.98) | 1 (2.07) | 0 (0.00) | 0 (0.00) | 0 (0.00) | 0 (0.00) | 0 (0.00) | NA | -3.74 (-7.84, 0.48) | 0.078 |
| **sPTB-noPPROM** | | | | | | | | | | | | | | | | | | | | | | | | | | | | |
| All preterm birth | 48 (17.45) | 7 (48.28) | 0 (0.00) | 1 (9.35) | 2 (19.61) | 4 (31.25) | 4 (30.08) | 1 (7.19) | 2 (15.63) | 2 (20.62) | 4 (25.97) | 0 (0.00) | 6 (30.46) | 0 (0.00) | 1 (7.87) | 0 (0.00) | 2 (15.63) | 4 (29.41) | 5 (43.10) | 0 (0.00) | 1 (14.49) | 0 (0.00) | 2 (44.44) | 0 (0.00) | 0 (0.00) | -100.00 | -1.66 (-8.10, 5.01) | 0.579 |
| Extreme preterm birth | 40 (157.48) | 5 (357.14) | 0 (0.00) | 1 (166.67) | 0 (0.00) | 4 (222.22) | 4 (285.71) | 0 (0.00) | 2 (285.71) | 2 (181.82) | 4 (333.33) | 0 (0.00) | 4 (250.00) | 0 (0.00) | 1 (83.33) | 0 (0.00) | 2 (125.00) | 4 (190.48) | 5 (277.78) | 0 (0.00) | 1 (166.67) | 0 (0.00) | 1 (111.11) | 0 (0.00) | 0 (0.00) | NA | -1.58 (-6.85, 4.07) | 0.549 |
| Very preterm birth | 5 (10.85) | 2 (125.00) | 0 (0.00) | 0 (0.00) | 0 (0.00) | 0 (0.00) | 0 (0.00) | 1 (45.45) | 0 (0.00) | 0 (0.00) | 0 (0.00) | 0 (0.00) | 1 (29.41) | 0 (0.00) | 0 (0.00) | 0 (0.00) | 0 (0.00) | 0 (0.00) | 0 (0.00) | 0 (0.00) | 0 (0.00) | 0 (0.00) | 1 (125.00) | 0 (0.00) | 0 (0.00) | -100.00 | -3.47 (-7.26, 3.48) | 0.267 |
| Moderate preterm birth | 3 (5.85) | 0 (0.00) | 0 (0.00) | 0 (0.00) | 2 (111.11) | 0 (0.00) | 0 (0.00) | 0 (0.00) | 0 (0.00) | 0 (0.00) | 0 (0.00) | 0 (0.00) | 1 (23.26) | 0 (0.00) | 0 (0.00) | 0 (0.00) | 0 (0.00) | 0 (0.00) | 0 (0.00) | 0 (0.00) | 0 (0.00) | 0 (0.00) | 0 (0.00) | 0 (0.00) | 0 (0.00) | NA | 1.69 (-3.20, 6.59) | 0.435 |
| Late preterm birth | 0 (0.0) | 0 (0.00) | 0 (0.00) | 0 (0.00) | 0 (0.00) | 0 (0.00) | 0 (0.00) | 0 s(0.00) | 0 (0.00) | 0 (0.00) | 0 (0.00) | 0 (0.00) | 0 (0.00) | 0 (0.00) | 0 (0.00) | 0 (0.00) | 0 (0.00) | 0 (0.00) | 0 (0.00) | 0 (0.00) | 0 (0.00) | 0 (0.00) | 0 (0.00) | 0 (0.00) | 0 (0.00) | NA | NA | NA |
| **PTB-PPROM** | | | | | | | | | | | | | | | | | | | | | | | | | | | | |
| All preterm birth | 39 (9.35) | 1 (17.86) | 1 (12.50) | 0 (0.00) | 3 (29.13) | 2 (17.39) | 2 (20.83) | 3 (19.74) | 4 (20.20) | 0 (0.00) | 1 (3.98) | 2 (8.20) | 5 (17.01) | 0 (0.00) | 0 (0.00) | 2 (8.26) | 0 (0.00) | 0 (0.00) | 2 (9.26) | 4 (20.00) | 1 (4.81) | 2 (13.70) | 2 (19.61) | 1 (10.64) | 1 (8.77) | -50.88 | 3.88 (-45.79, 100.66) | 0.922 |
| Extreme preterm birth | 24 (132.60) | 0 (0.00) | 0 (0.00) | 0 (0.00) | 2 (400.00) | 2 (400.00) | 1 (1000.00) | 1 (125.00) | 3 (375.00) | 0 (0.00) | 0 (0.00) | 1 (83.33) | 4 (250.00) | 0 (0.00) | 0 (0.00) | 2 (200.00) | 0 (0.00) | 0 (0.00) | 0 (0.00) | 4 (666.67) | 0 (0.00) | 2 (166.67) | 0 (0.00) | 1 (100.00) | 1 (71.43) | NA | 8.77 (-73.29, 313.23) | 0.904 |
| Very preterm birth | 8 (13.29) | 1 (62.50) | 0 (0.00) | 0 (0.00) | 1 (71.43) | 0 (0.00) | 0 (0.00) | 0 (0.00) | 0 (0.00) | 0 (0.00) | 1 (31.25) | 0 (0.00) | 1 (21.28) | 0 (0.00) | 0 (0.00) | 0 (0.00) | 0 (0.00) | 0 (0.00) | 2 (66.67) | 0 (0.00) | 1 (29.41) | 0 (0.00) | 1 (71.43) | 0 (0.00) | 0 (0.00) | -100.00 | -1.32 (-49.94, 95.23) | 0.930 |
| Moderate preterm birth | 5 (5.43) | 0 (0.00) | 1 (71.43) | 0 (0.00) | 0 (0.00) | 0 (0.00) | 1 (37.04) | 1 (27.78) | 1 (15.38) | 0 (0.00) | 0 (0.00) | 0 (0.00) | 0 (0.00) | 0 (0.00) | 0 (0.00) | 0 (0.00) | 0 (0.00) | 0 (0.00) | 0 (0.00) | 0 (0.00) | 0 (0.00) | 0 (0.00) | 1 (45.45) | 0 (0.00) | 0 (0.00) | NA | -1.74 (-5.75, 2.40) | 0.369 |
| Late preterm birth | 2 (0.81) | 0 (0.00) | 0 (0.00) | 0 (0.00) | 0 (0.00) | 0 (0.00) | 0 (0.00) | 1 (10.42) | 0 (0.00) | 0 (0.00) | 0 (0.00) | 1 (7.52) | 0 (0.00) | 0 (0.00) | 0 (0.00) | 0 (0.00) | 0 (0.00) | 0 (0.00) | 0 (0.00) | 0 (0.00) | 0 (0.00) | 0 (0.00) | 0 (0.00) | 0 (0.00) | 0 (0.00) | NA | -2.16 (-4.64, 0.45) | 0.094 |
| **iPTB** | | | | | | | | | | | | | | | | | | | | | | | | | | | | |
| All preterm birth | 53 (8.23) | 1 (6.62) | 2 (14.71) | 1 (6.58) | 2 (14.93) | 1 (5.43) | 2 (9.30) | 1 (5.78) | 2 (6.64) | 4 (19.80) | 3 (11.58) | 4 (14.23) | 0 (0.00) | 3 (8.04) | 2 (6.54) | 1 (2.62) | 5 (15.72) | 3 (8.00) | 3 (7.33) | 4 (9.55) | 3 (7.43) | 2 (6.35) | 1 (5.03) | 2 (9.05) | 1 (3.77) | -43.02 | -0.95 (-38.08, 58.77) | 0.887 |
| Extreme preterm birth | 22 (205.61) | 1 (500.00) | 1 (500.00) | 0 (0.00) | 1 (500.00) | 0 (0.00) | 0 (0.00) | 0 (0.00) | 1 (250.00) | 0 (0.00) | 2 (1000.00) | 1 (166.67) | 0 (0.00) | 3 (500.00) | 1 (250.00) | 1 (166.67) | 2 (666.67) | 0 (0.00) | 2 (200.00) | 1 (250.00) | 1 (125.00) | 1 (166.67) | 0 (0.00) | 2 (333.33) | 1 (166.67) | -66.67 | 49.95 (-29.19, 208.44) | 0.302 |
| Very preterm birth | 12 (20.24) | 0 (0.00) | 1 (83.33) | 0 (0.00) | 0 (0.00) | 0 (0.00) | 0 (0.00) | 1 (50.00) | 0 (0.00) | 2 (80.00) | 0 (0.00) | 1 (27.78) | 0 (0.00) | 0 (0.00) | 1 (38.46) | 0 (0.00) | 1 (35.71) | 2 (76.92) | 0 (0.00) | 1 (24.39) | 0 (0.00) | 1 (34.48) | 1 (71.43) | 0 (0.00) | 0 (0.00) | NA | 30.95 (-44.33, 208.12) | 0.560 |
| Moderate preterm birth | 8 (8.17) | 0 (0.00) | 0 (0.00) | 0 (0.00) | 1 (38.46) | 0 (0.00) | 0 (0.00) | 0 (0.00) | 1 (16.39) | 1 (29.41) | 1 (17.86) | 0 (0.00) | 0 (0.00) | 0 (0.00) | 0 (0.00) | 0 (0.00) | 0 (0.00) | 1 (17.86) | 0 (0.00) | 1 (15.15) | 2 (41.67) | 0 (0.00) | 0 (0.00) | 0 (0.00) | 0 (0.00) | NA | -0.56 (-4.50, 3.42) | 0.750 |
| Late preterm birth | 11 (2.31) | 0 (0.00) | 0 (0.00) | 1 (9.01) | 0 (0.00) | 1 (9.09) | 2 (12.99) | 0 (0.00) | 0 (0.00) | 1 (7.41) | 0 (0.00) | 2 (10.26) | 0 (0.00) | 0 (0.00) | 0 (0.00) | 0 (0.00) | 2 (7.69) | 0 (0.00) | 1 (3.15) | 1 (3.25) | 0 (0.00) | 0 (0.00) | 0 (0.00) | 0 (0.00) | 0 (0.00) | NA | -5.16 (-9.32, -1.05) | 0.019 |
